# Effects of arbuscular mycorrhizal fungi on the reduction of arsenic accumulation in plants: a meta-analysis

**DOI:** 10.3389/fpls.2024.1327649

**Published:** 2024-04-05

**Authors:** Shangyan Hao, Ye Tian, Zhiqing Lin, Linzhi Xie, Xinbin Zhou, Gary S. Bañuelos

**Affiliations:** ^1^ College of Resources and Environment, Southwest University, Chongqing, China; ^2^ Department of Environmental Sciences, Southern Illinois University, Edwardsville, IL, United States; ^3^ Department of Biological Sciences, Southern Illinois University, Edwardsville, IL, United States; ^4^ Agricultural Research Service, United States Department of Agriculture, Parlier, CA, United States

**Keywords:** arbuscular mycorrhizal fungi, arsenic, reduction, factors, meta-analysis

## Abstract

Arsenic (As) accumulation in plants is a global concern. Although the application of arbuscular mycorrhizal fungi (AMF) has been suggested as a potential solution to decrease As concentration in plants, there is currently a gap in a comprehensive, quantitative assessment of the abiotic and biotic factors influencing As accumulation. A meta-analysis was performed to quantitatively investigate the findings of 76 publications on the impacts of AMF, plant properties, and soil on As accumulation in plants. Results showed a significant dose-dependent As reduction with higher mycorrhizal infection rates, leading to a 19.3% decrease in As concentration. AMF reduced As(V) by 19.4% but increased dimethylarsenic acid (DMA) by 50.8%. AMF significantly decreased grain As concentration by 34.1%. AMF also improved plant P concentration and dry biomass by 33.0% and 62.0%, respectively. The most significant reducing effects of As on AMF properties were seen in single inoculation and experiments with intermediate durations. Additionally, the benefits of AMF were significantly enhanced when soil texture, soil organic carbon (SOC), pH level, Olsen-P, and DTPA-As were sandy soil, 0.8%–1.5%, ≥7.5, ≥9.1 mg/kg, and 30–60 mg/kg, respectively. AMF increased easily extractable glomalin-related soil protein (EE-GRSP) and total glomalin-related soil protein (T-GRSP) by 23.0% and 28.0%, respectively. Overall, the investigated factors had significant implications in developing AMF-based methods for alleviating the negative effects of As stress on plants.

## 1 Introduction

Arsenic (As) contamination in agricultural soils is a common environmental issue that poses remarkable risks to human health and ecosystem sustainability (Wan et al., 2024). As a toxic metalloid, As can naturally occur in soils or be introduced through various human activities such as mining, industrial processes, and application of arsenical pesticides (Mawia et al., 2021; Gui et al., 2023). As contamination is a global issue in countries such as the United States, China, Argentina, Australia, Bangladesh, Chile, India, Mexico, Thailand, and Vietnam (Baruah et al., 2021; Wang et al., 2023; Jahandari and Abbasnejad, 2024). The As pollution in China is of grave concern, with the nation’s farmland soil arsenic levels averaging a concerning 11.83 mg/kg, significantly surpassing the global average of 7.20 mg/kg (Ren et al., 2022). As uptake by plant roots and its subsequent translocation to edible parts of plants can result in potential health risks throughout the food chain. According to the U.S. Agency for Toxic Substances and Disease Registry (ATSDR), As was classified as a top hazardous substance in the United States (Escudero-Lourdes, 2016). As exposure has been found to contribute to different cancer types such as skin, lung, liver, kidney, and bladder cancers (Zakir et al., 2022). Worldwide, the threat of As poisoning affects over 230 million people, with 180 million in Asia being especially vulnerable (Shaji et al., 2021). Hence, there is a growing attention to research on soil As pollution issues among researchers.

Arbuscular mycorrhizal fungi (AMF) are beneficial soil microorganisms, which form mutualistic symbiotic associations with roots in most land plant species (Hawkins et al., 2023). These symbiotic relationships play crucial roles in enhancing plant nutrient uptake, stress tolerance, and overall growth and development. Recently, several research works have highlighted the key role of AMF in the absorption, translocation, and accumulation of As in plants (Li et al., 2016; Alam et al., 2020; Mitra et al., 2022). AMF involvement may be crucial in decreasing As phytoavailability, which achieves this by stabilizing As through mycelium and glomalin (Wu et al., 2015; Zhang et al., 2020; Riaz et al., 2021). AMF mycelium forms a network in the soil, effectively immobilizing and trapping As to prevent its uptake by plants. In addition, glomalin, a glycoprotein produced by AMF, binds to and sequesters As to further reduce its availability for plant uptake (Maldonado-Mendoza and Harrison, 2018). These mechanisms, which are adopted by AMF to stabilize As, significantly contribute to lowering its potential impact on plants, enhancing agricultural system sustainability, and mitigating the risks associated with As contamination.

The interaction of AMF colonization and As accumulation holds great promise for ensuring safe food production and implementing bioremediation programs (Gupta et al., 2022). AMF inoculation effect on As concentration in soil–plant systems has been extensively studied by scholars all over the world. Singh et al. (2023) found that applying 10 g/kg *Glomus mosseae* significantly reduced arsenic in wheat grains (Singh et al., 2023). Gupta et al. (2021) reported that *Rhizophagus intraradices* inoculation counteracted As-induced changes in sugar metabolism, affecting enzyme activities related to starch phosphorylase, α-amylase, β-amylase, acid invertase, sucrose synthase, and sucrose phosphate synthase in leaves (Gupta et al., 2021). Under As stress of 25 mg/kg, the synergistic effect of *Funneliformis mosseae* and iron can reduce the toxic effects of arsenic by enhancing phosphorus uptake and increasing the activity of antioxidant enzymes in rice (Zhou et al., 2023). However, a comprehensive review of previous experimental studies revealed significant inconsistencies in the published results. Some research works have reported that AMF inoculation resulted in significant As concentration reductions in both roots and shoots of maize (Yu et al., 2009), while others have found increases in As concentration in maize roots (Long et al., 2021). Similarly, conflicting results have been reported in rice, with some research works showing no significant effect of AMF inoculation on total As concentration in rice grains (Zhang, X. et al., 2016), while some others have reported increased total and inorganic As concentrations in rice grains (Li et al., 2016). Several qualitative literature reviews have focused on the complex interaction frameworks of environmental and biological factors influencing AMF-mediated As concentration (Cabral et al., 2015; Neidhardt, 2021; Mitra et al., 2022; Tan et al., 2023). However, no comprehensive meta-analysis has been performed to quantitatively determine and evaluate the relationship between AMF and As concentration in plants under various environmental and biological conditions. Meta-analysis is a powerful method aimed at deriving broad generalizations by pooling and analyzing outcomes from multiple studies. Its fundamental goal is to provide a more comprehensive understanding than what can be obtained from individual primary studies alone. However, it is important to note that meta-analysis has limitations. The quality of each study included and the potential publication bias can influence the results of the meta-analysis. Additionally, the inclusion of new studies in the future may lead to updates and modifications in the conclusions of the meta-analysis. It is a dynamic process that requires continuous evaluation and consideration of new evidence (Gurevitch et al., 2018). Therefore, this research aims to 1) verify the impact of AMF inoculation on As accumulation in plants grown in As-contaminated soils; 2) quantify the effects of AMF on plant As concentration, considering various biotic factors (e.g., AMF root colonization rate, AMF inoculum type, AMF species, and plant family) and abiotic factors [e.g., soil texture, soil pH, and soil organic carbon (SOC)]; and 3) investigate the mechanisms supported by data as proposed in the literature. Additionally, potential future research directions regarding the interaction between AMF and As will be discussed.

## 2 Materials and methods

### 2.1 Bibliometric analysis using CiteSpace

CiteSpace is a new statistical analysis software widely used for bibliometric analysis and visualization in the academic community (Chen and Song, 2019). A systematic literature search was conducted from the Web of Science (WoS) Core Collection database on the topic of arsenic and arbuscular mycorrhizal fungi. A total of 227 publications from 1994 to 2024 were collected from the database and used CiteSpace software to visualize and analyze various aspects including annual publication volume, research countries, and keywords in this field (**Supplementary Figure S1**). The scientific knowledge mapping visualized by CiteSpace facilitated a better understanding of theoretical terms and provided some background knowledge for our meta-analysis work.

### 2.2 Literature collection

**Figure 1:**
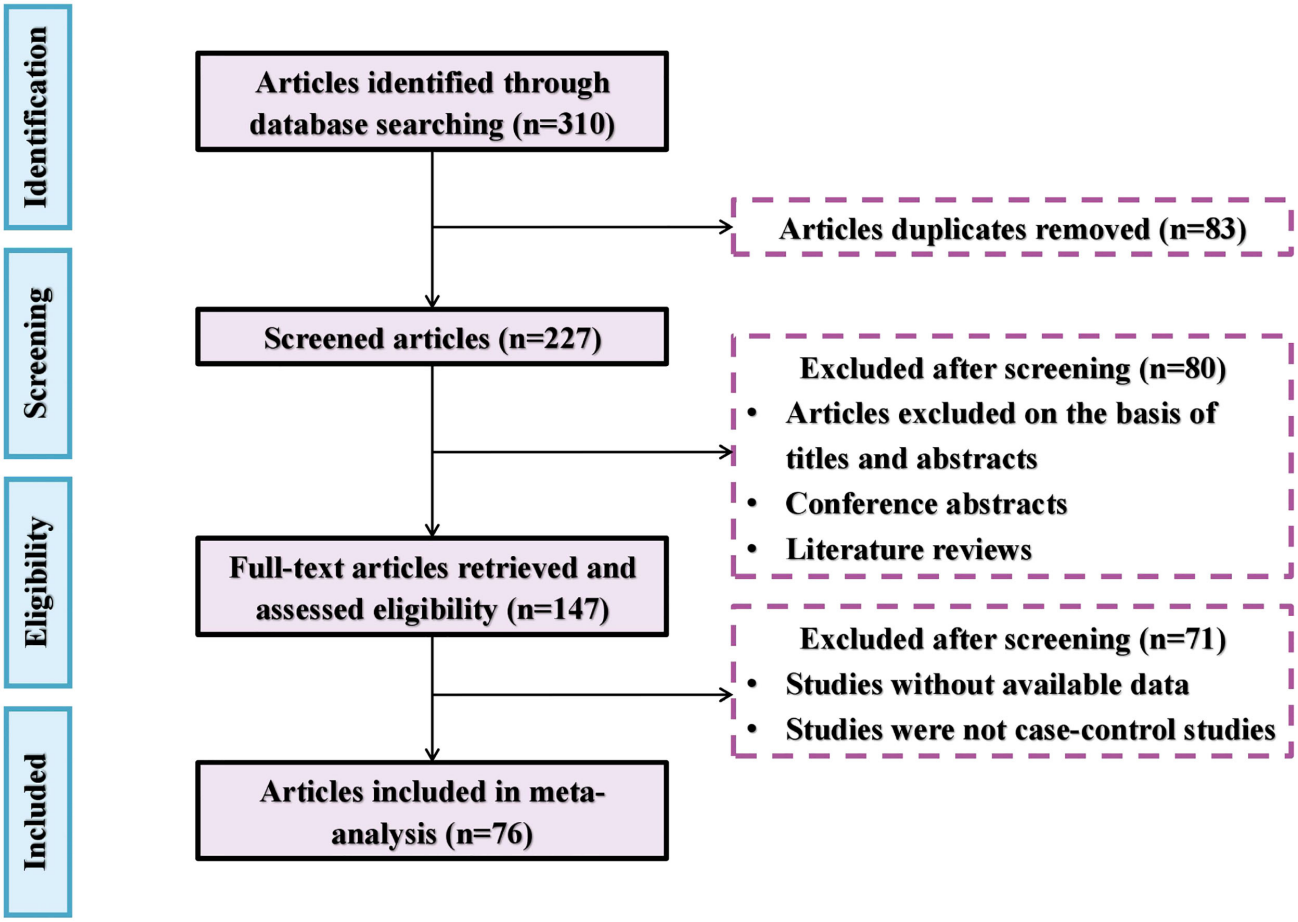
The flowchart of the literature selection procedure.

**Figure 2:**
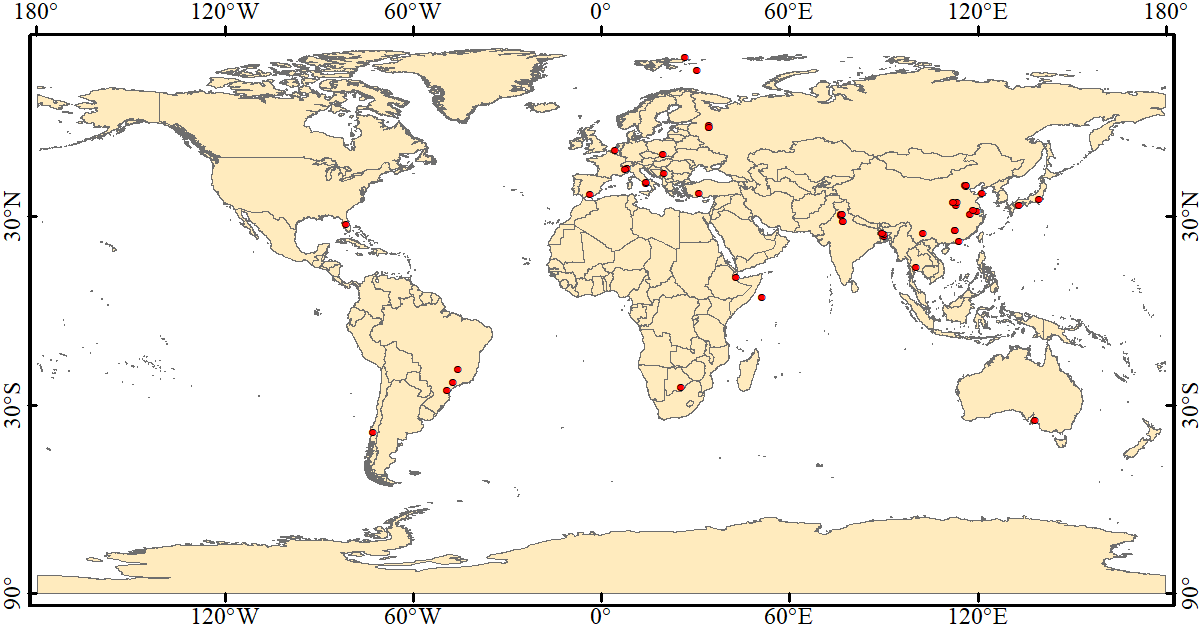
Geographical distribution of 76 published research articles in the world.

Peer-reviewed research papers on the subject were identified through the Web of Science (https://apps.webofknowledge.com) as of the end of December 2023. Search keywords included *(As or arsenic or arsenite or arsenate) and (plant or crop) and (Arbuscular mycorrhizal fungi or AM or AMF) and (soil)*. The selected research articles for meta-analysis needed to meet the following criteria: 1) the research must be original and focus on plant performance in As-contaminated soils in response to AMF treatments. 2) The experiment must include both a control group (i.e., without AMF application) and a treatment group (i.e., with AMF application). 3) The research paper must include the mean and standard deviation values of As concentration in different tissues of plants. If no standard deviation was reported, one-tenth of the mean value was used instead (Luo and Zhang, 2006). 4) Each treatment and the control must have at least three replicates. 5) The experiment was conducted using sterilized soil or sand mixture. 6) The effect of AMF was determined in the absence of rhizobia so that the effect of nitrogen-fixing bacteria was eliminated. The literature selection procedure for the meta-analysis is shown in **Figure 1**, including a total of 76 research articles with 1,362 observations from different parts of the world (**Figure 2**).

Data in numerical form were extracted directly from the original literature or tables, and data in graphical form were extracted using WebPlotDigitizer 4.5 (https://apps.automeris.io/wpd/index.zh_CN.html). The extracted data were used to establish a database using Microsoft Excel 2016, including the following categories: authors, titles, published journals, publication date, plant species, AMF species, soil physical and chemical properties, P fertilizer application and the application rate, As concentration, dry weight, and P concentration and its standard deviation in different plant tissues. In the case of reporting a standard error (SE), the standard deviation (SD) is calculated by SD = SE * $\sqrt{\text{n}}$ .

### 2.3 Categorical independent variables

The effects of AMF on As concentration in different plant tissues were determined, including grain, husk, straw, shoot, and root tissues. To gain a better and more comprehensive understanding of how soil biotic and abiotic factors impact the response of selected variables to the experimental treatments, we classified those factors into 10 groups. These groups include both biotic factors (such as AMF inoculum type, AMF species, plant family, and experimental duration) and abiotic factors (such as soil texture, pH, SOC, soil DTPA-As, chemical forms of As, and soil Olsen-P levels).

The AMF inoculum type was classified into two categories: single and mixed AMF species. Single inoculation entailed using only one AMF species in the experiment, while mixed inoculation involved using multiple AMF species obtained from either field extraction or commercial suppliers. The species of AMF were classified into *Rhizophagus irregularis*, *R. intraradices*, *G. mosseae*, *Glomus intraradices*, *Glomus caledonium*, *Glomus* sp., *Glomus geosporum*, *Glomus versiforme*, *Glomus etunicatum*, and *F. mosseae*. Plant families were divided into Leguminosae, Pteridaceae, Compositae, Poaceae, Solanaceae, Plantaginaceae, and Melastomataceae. The experiment durations were categorized into three groups based on Lehmann and Rillig (2015): short with<56 days, medium with 56–112 days, and long with ≥112 days. According to the USDA Soil Classification (soils.USDA.gov), the soil texture included sandy and non-sandy soils. Sandy soil had a sand content of ≥50%. The soil pH had three levels: acid (pH< 6.5), neutral (pH 6.5–7.4), and alkaline (pH ≥ 7.4). The SOC also had three levels: low (SOC< 0.8%), medium (SOC 0.8–1.5%), and high (SOC ≥ 1.5%). Soil Olsen-P used as the background value was classified into two categories: deficiency (<9 mg/kg) and non-deficiency (≥9.1 mg/kg). P fertilizer application level was classified into three categories: low (<30 mg/kg), medium (30–60 mg/kg), and high (≥60 mg/kg). Soil DTPA-As used as the background value was divided into three categories: low (soil with final As concentration of<20 mg/kg or As added in irrigation water of<0.2 mg/L), medium (20–50 mg/kg or 0.2–1.0 mg/L), and high (≥50 mg/kg or ≥1.0 mg/L). The species of exogenous As were divided into As(III) and As(V). These categorization methods were used based on Lehmann and Rillig (2015) and Qiu et al. (2022).

### 2.4 Statistical analysis

The natural logarithm response ratio (ln*R*) method was used to calculate the effect size and variance of each set of data using MetaWin 2.1.


$${\text{ln}R=\text{ln}(X}_{E}{\mathrm{/X}}_{C}),$$

$$v=\frac{SD_{E}^{2}}{n_{E}X_{E}^{2}}+\frac{SD_{C}^{2}}{n_{C}X_{C}^{2}}$$

where ln*R* is the natural logarithm of the response ratio, defined as the effect size, *X_E_* is the averaged plant As concentration of the treatment group, *X_C_* is the averaged plant As concentration of the control group, and *n_E_* and *n_C_* are the number of replicates in the treatment group and the control group, respectively. *SD_E_* and *SD_C_* are the standard deviations of the treatment group and the control group, respectively.

To assess the overall variability of each variable, the Q_T_ statistic and the I^2^ index were used (**Supplementary Table S1**). If the included studies showed significant heterogeneity (*p*< 0.05), either a random-effects model (REM) or a fixed-effects model (FEM) was applied for analysis. Since our dataset had a p-value below 0.05, the appropriate choice for analysis was the REM. The entire dataset was then divided into different subgroups to investigate the source of heterogeneity. Before the weighted analysis, the software Origin 2019 was used to generate a frequency distribution histogram of ln*R* and fit the Gaussian function to determine the normality of the data (**Supplementary Figure S2**). The bootstrap method was employed to establish the 95% confidence intervals (CIs) for effect sizes across all study variables and categories. The percentage change in response was calculated as (R − 1) × 100%. The positive effect size and positive percentage change indicated that AMF inoculation promoted plant As accumulation, and the negative effect size and negative percentage change indicated that AMF inhibited plant As accumulation. Publication bias is a common issue in meta-analyses due to the tendency to not publish non-significant results. This study aimed to address this problem by utilizing two methods to evaluate potential publication bias. One method used was the calculation of Rosenthal’s fail-safe number using MetaWin 2.1. A fail-safe number equal to or greater than 5 * N + 10 (N representing the number of case studies) suggests a robust result. Another method employed was the use of funnel plots, where effect sizes were plotted against sample variances for ln*R* to assess publication bias. The data analysis results were then visually presented as color maps using Origin 2019 and as forest plots using GraphPad Prism 9.0.

## 3 Results

### 3.1 Effects of AMF on As concentrations, P/As ratio, and dry biomass in different tissues of plants

**Figure 3:**
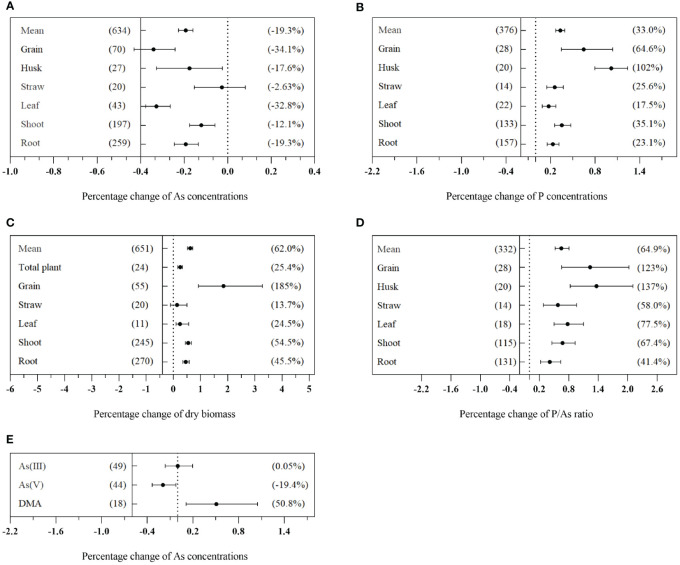
Effects of arbuscular mycorrhizal fungi (AMF) inoculation on total As concentration **(A)**, P concentration **(B)**, dry weight **(C)**, and P/As ratio **(D)** in different tissues of plants and different valence As concentration **(E)** in plants. The value in the left bracket is the number of tests included in the analysis, and the value in the right bracket represents the size of the effect value. When the mean value is regular, there is a positive effect, and when it is negative, there is a negative effect. When CI does not overlap with 0, there is no significant effect. The same as below.

The effects of AMF on As concentrations in various tissues of plants grown on As-contaminated soil are illustrated in **Figure 3**. In comparison to non-mycorrhizal plants, those inoculated with AMF exhibited a significant reduction in total As concentration, with an average decrease of 19.3% (CI: −22.6% to −16.0%) (**Figure 3A**). Additionally, AMF inoculation resulted in an improvement in the nutritional status of plants, as evidenced by a mean increase of 33.0% (CI: 27.0% to 39.2%) in P concentration, 62.0% (CI: 52.1% to 71.7%) in dry biomass, and 64.9% (CI: 53.3% to 80.5%) in the P/As ratio (**Figures 3B–D**). The impact of AMF on the concentration of As in plants varied significantly among different plant tissues (Q_b_ = 246, *p*< 0.01). There was a significant decrease in As concentration in grains, husks, leaves, shoots, and roots, with reduction rates of 34.1% (CI: −43.1% to −24.2%), 17.6% (CI: −32.8% to −2.5%), 32.8% (CI: −37.7% to −26.4%), 12.1% (CI: −17.6% to −5.8%), and 19.3% (CI: −24.5% to −13.5%), respectively. However, there was no significant effect on As concentration in straws (**Figure 3A**). It is worth noting that AMF inoculation significantly increased the grain dry biomass by 185% (CI: 91.9% to 328%) (**Figure 3C**). The application of AMF resulted in a significant reduction of As(V) concentration by 19.4% (CI: −33.4% to −2.34%), while no significant effect was observed on the concentration of As(III). Moreover, AMF application led to a significant increase in dimethylarsenic acid (DMA) concentration by 50.8% (CI: 11.0% to 115%) (**Figure 3E**).

### 3.2 Effects of abiotic factors on As concentration in plants treated with AMF

**Figure 4:**
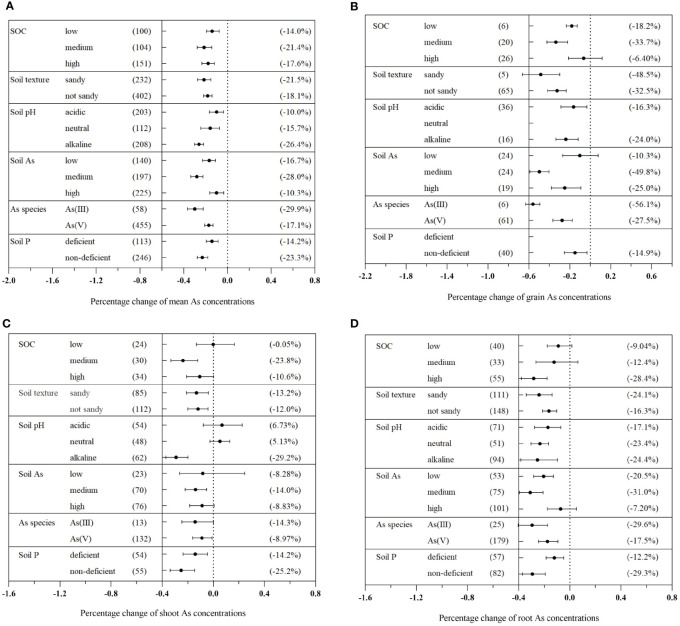
Effects of different soil properties on As concentration in the total plants **(A)**, grains **(B)**, shoots **(C)**, and roots **(D)** with arbuscular mycorrhizal fungi (AMF) inoculation.

The impact of AMF on plant As concentration varied based on the soil properties (**Figure 4**). Medium SOC content (0.8%–1.5%) exhibited the most significant reduction in plant As concentration, with a decrease of 21.4% (CI: −27.7% to −14.2%) (**Figure 4A**). In terms of specific plant tissues, medium SOC levels (0.8%–1.5%) were found to significantly decrease As concentration in grains, shoots, and roots (**Figures 4B–D**). Sandy soils exhibited a higher reduction in plant As concentration compared to non-sandy soils, with reductions of 21.5% (CI: −27.4% to −15.0%) and 18.1% (CI: −21.8% to −14.1%), respectively (**Figure 4A**). The effect of varying soil pH levels on As concentration was determined to be significant (Q_b_ = 31.5, *p*< 0.01). Alkaline soil was identified as the most effective in reducing As concentration in different plant tissues mediated by AMF. Overall, there was a reduction in As concentration of 26.4% (CI: −30.1% to −21.9%) in total plants, 24.0% (CI: −33.7% to −11.8%) in grains, 29.2% (CI: −37.2% to −19.9%) in shoots, and 24.4% (CI: −38.4% to −9.64%) in roots (**Figures 4A–D**).

Significant differences were observed among different levels of soil As concentration (Q_b_ = 95.1, *p*< 0.01). Medium As concentration in the soil significantly inhibited As concentration in different plant tissues treated with AMF, resulting in reduction of 28.0% (CI: −33.5% to −22.4%) in total plants, 49.8% (CI: −59.1% to −40.3%) in grains, 14.0% (CI: −22.1% to −5.17%) in shoots, and 31.0% (CI: −39.5% to −21.0%) in roots (**Figures 4A–D**). Furthermore, significant inhibitory effects on As concentration were observed in soils containing deficient Olsen-P, with an average decrease of 23.3% (CI: −27.9% to −18.4%) (**Figure 4A**). Similarly, various abiotic factors influenced the impact of AMF on plant P concentration and dry biomass (**Supplementary Figures S4**, **S6**).

### 3.3 Effects of biotic factors on As concentration in plants inoculated with AMF

**Figure 5:**
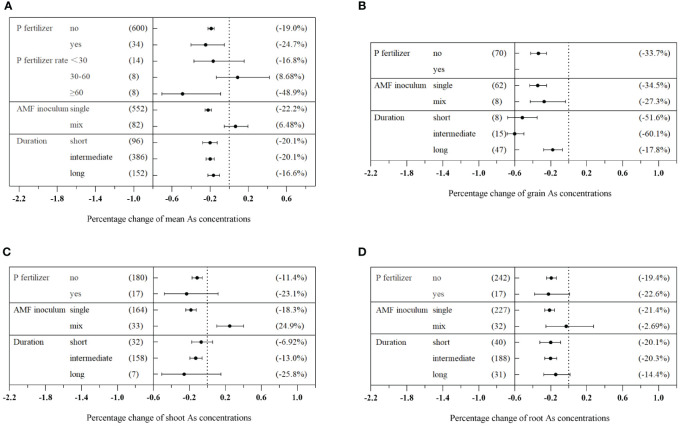
Effects of different external factors on As concentrations in the total plants **(A)**, grains **(B)**, shoots **(C)**, and roots **(D)** with arbuscular mycorrhizal fungi (AMF) inoculation.

**Figure 6:**
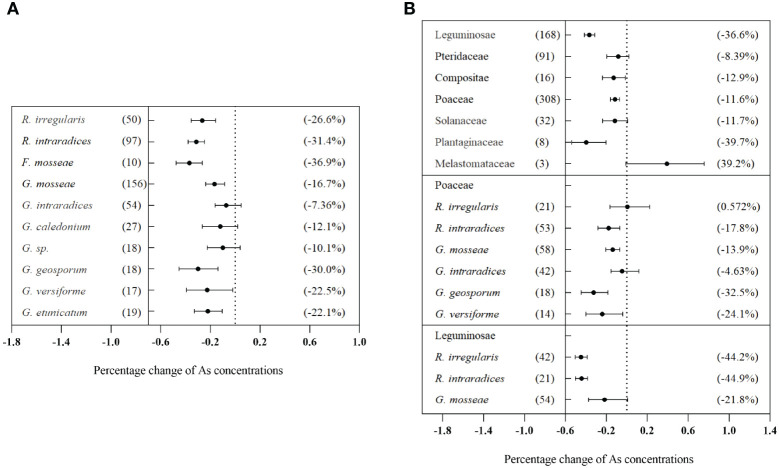
Effects of different arbuscular mycorrhizal fungi (AMF) species on As concentrations in total plants **(A)** and effects of AMF inoculation on As concentration in different plant families **(B)**.

Significant differences were observed in the effects of different inoculation methods on plant As concentration (Q_b_ = 211, *p*< 0.01). Single inoculation with AMF significantly decreased As concentration by 22.2% (CI: −25.3% to −18.8%). However, mixed inoculation showed a non-significant increase of 6.48% (CI: −5.33% to 19.4%) (**Figure 5A**). Moreover, there were significant differences in the effects of different AMF species on plant As concentration (Q_b_ = 226, *p*< 0.01). Inoculation of *F. mosseae* and *R. intraradices* reduced As concentration by more than 30%, which was superior to other AMF species (**Figure 6A**). Additionally, the effects of AMF inoculation on As concentration in plants varied significantly among different families (Q_b_ = 537, *p*< 0.01). In Leguminosae plants, the inoculation with *R. intraradices* resulted in the greatest reduction in As concentration (−44.9%, CI: −50.4% to −38.7%) (**Figure 6B**). The duration of the experiment did not have a significant effect on As concentration in plants (Q_b_ = 6.78, *p* = 0.568) (**Figure 5A**). However, intermediate experiment durations significantly decreased As concentration in different plant tissues treated with AMF. This result led to a reduction of 60.1% (CI: −68.3% to −49.8%) in grains, 13.0% (CI: −19.6% to −5.96%) in shoots, and 20.3% (CI: −26.6% to −13.4%) in roots (**Figures 5B–D**). Similarly, various biotic factors influenced the impact of AMF on plant P concentration and dry biomass (**Supplementary Figures S5**, **S7**).

**Figure 7:**
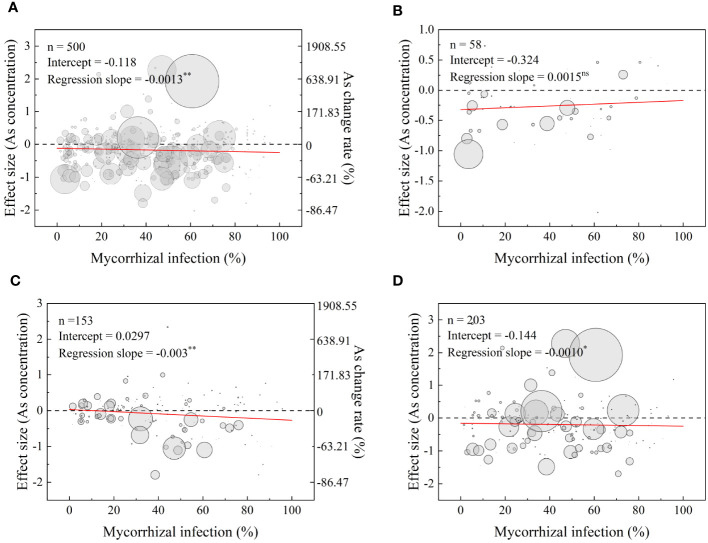
Relationships between mycorrhizal infection rate and effect size (As concentration) in plants **(A)**, grains **(B)**, shoots **(C)**, and roots **(D)**. The red lines represent the prediction of the meta-regression model. n: number of points. Significance of the model: ns, not significant; *significant at *p*< 0.05; **significant at *p*< 0.01. The right scales shown on **(A, C)** represent the values equivalent to the effect size in percentage of As concentrations change rates.

The meta-regression model results for the full dataset revealed a dose-dependent decrease in As concentrations in plants as the mycorrhizal infection rate increased (**Figure 7A**). Specifically, at mycorrhizal infection rates of 34.2% and 80.9%, the total As concentration in the plants decreased by 15% and 20%, respectively. The analysis also indicated a significant decrease in As concentration in shoots and roots as the mycorrhizal infection rate increased (**Figures 7C, D**). However, there were no significant impacts observed on the As concentration in grains (**Figure 7B**). Furthermore, the analysis demonstrated a significant linear positive effect of mycorrhizal infection rate on the effect size of P concentration and dry biomass (**Supplementary Figures S8**, **S9**).

### 3.4 Effect of AMF treatment on As concentration in plants with P fertilizer

The study observed the influence of P fertilizer on the inhibitory effects of AMF on plant As accumulation. In the absence of P fertilizer, a significant reduction of 19.0% (CI: −22.3% to −15.8%) in plant As concentration was observed due to AMF. However, the presence of P fertilizer enhanced this reduction effect, resulting in a decrease of 24.7% (CI: −40.2% to −5.23%) in As concentration (**Figure 5A**). The effect of different P fertilizer conditions on plant As concentration showed a significant difference (Q_b_ = 14.7, *p*< 0.01). Under low P fertilizer conditions (<30 mg/kg), AMF decreased As concentration by 16.8% (CI: −37.0% to 15.3%), although the effect was not statistically significant. Interestingly, under high P fertilizer conditions (≥60 mg/kg), AMF significantly decreased As concentration by 48.9% (CI: −70.6% to −9.07%) (**Figure 5A**).

### 3.5 Effect of AMF treatment on As concentration and P/As ratio in plants under As exposure

**Figure 8:**
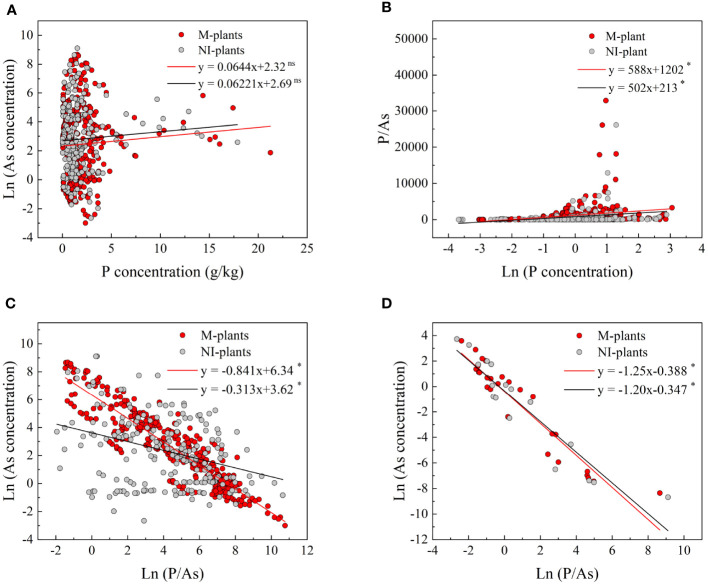
Relationships between P concentration and As concentration **(A)** and P/As ratio **(B)** in plants. Relationships between P/As ratio and As concentration in total plants **(C)** and grains **(D)**. Red dots represent mycorrhizal plants (M-plants), and gray dots represent non-mycorrhizal plants (NI-plants). ns, not significant; significant at *p< 0.05 and *p< 0.01.

The analysis of the fitting curve revealed that an increase in P concentration in plants resulted in a lower concentration of As in mycorrhizal plants compared to non-mycorrhizal plants (**Figure 8A**). Additionally, it was observed that the P/As ratio was higher in mycorrhizal plants compared to non-mycorrhizal plants (**Figure 8B**). These findings suggested that the AMF treatments enhanced the P accumulation and the P/As ratio in plants, consequently decreasing the As concentration. Furthermore, a significant negative correlation (*p*< 0.05) was observed between the P/As ratio and the As concentration in both total plants and grains (**Figures 8C, D**).

### 3.6 Effects of AMF on soil properties

**Figure 9:**
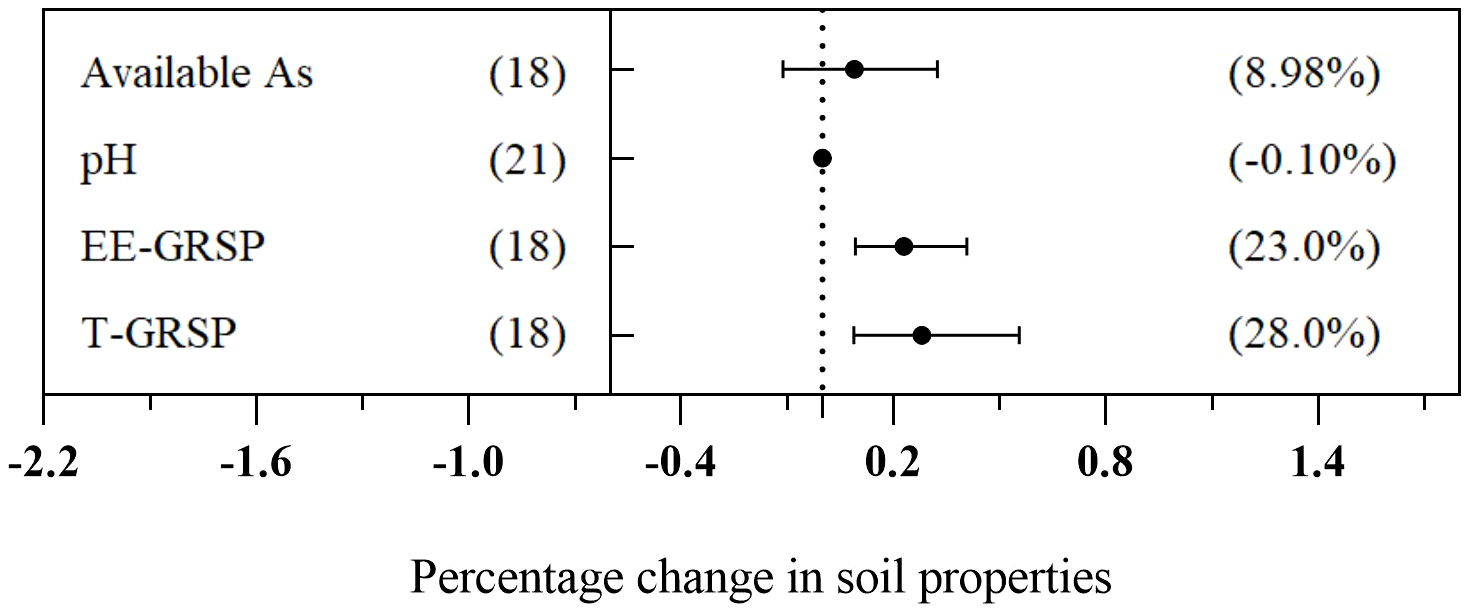
Change in soil properties induced by arbuscular mycorrhizal fungi (AMF) treatment.

To assess the changes in soil physical and chemical properties after AMF inoculation, we calculated the effect of soil pH, total As, easily extractable glomalin-related soil protein (EE-GRSP), and total glomalin-related soil protein (T-GRSP) in the AMF treatment group. The findings revealed that the AMF treatment had a significant effect on enhancing soil EE-GRSP and T-GRSP by 23.0% and 28.0%, respectively. However, there was no significant impact on the concentration of available As and soil pH (**Figure 9**).

### 3.7 Database and publication bias

From the extracted articles published by August 2023, a total of 76 articles met our selection criteria, and 1,362 data points were used for this meta-analysis. It was found that the fail-safe number was much higher than the threshold value (5 * N + 10, N is the number of case studies), indicating that these results were relatively robust (**Supplementary Table S1**). Additionally, the sample data points in the funnel plots were evenly distributed on both sides of the funnel, indicating the absence of publication bias (**Supplementary Figure S3**).

## 4 Discussion

### 4.1 How much does AMF treatment decrease total As concentration in plants and grains?

**Figure 10:**
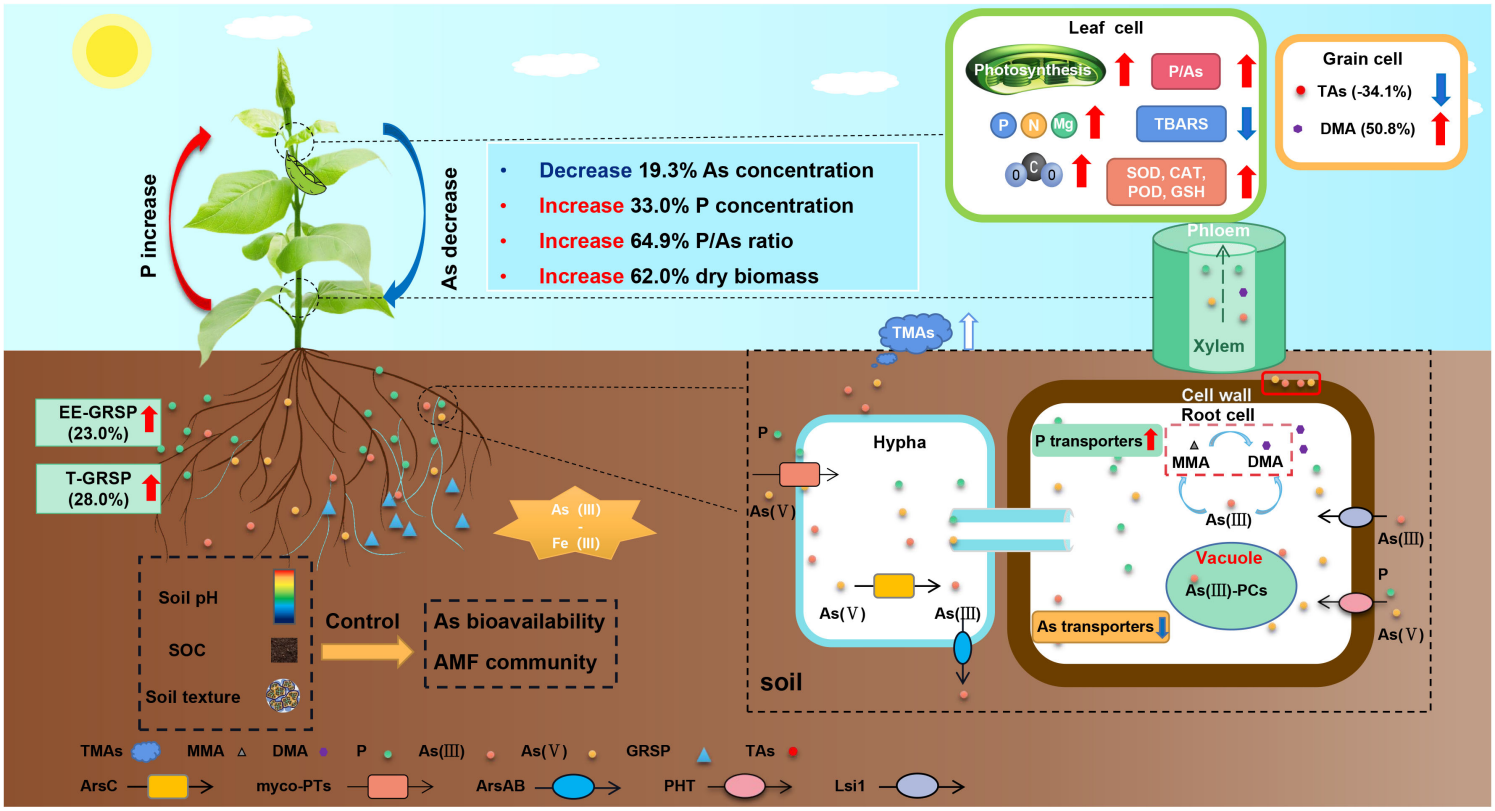
A conceptual flow diagram of the role of arbuscular mycorrhizal fungi (AMF) in regulating As bioavailability and accumulation in the plants. The red arrow represents an increase in content or effect, and the blue arrow represents a decrease.

The results of the meta-analyses performed on the entire dataset demonstrated significant and dose-dependent effects of AMF inoculation on decreasing As concentration in plants (**Figure 7A**). In addition, the analysis showed that AMF treatment significantly decreased the overall As concentration of plants by 19.3% and significantly reduced As concentration in grains by 34.1% (**Figure 3A**). These results revealed the potential of AMF inoculation as a promising strategy for the mitigation of As contamination in plants. A conceptual flow diagram outlining the AMF-induced reduction of As accumulation in plants has been specifically discussed in the last paragraph of the Discussion section (**Figure 10**). In fact, AMF reduced As accumulation while increasing P, N, K, Mg, Ca, Fe, Zn, Mn, Ni, and Se levels in grains, indicating that AMF may assist in overcoming mineral deficiencies in populations that consumed wheat-based diets, especially in As-contaminated areas (Gupta et al., 2022). AMF can play a key role in enhancing nutritional value and addressing mineral deficiencies in affected populations. Hence, AMF-based strategies were found to have the potential to provide multiple nutritional benefits in As-contaminated regions. Through the promotion of the availability and uptake of essential minerals, mycorrhizal colonization improved the nutritional quality of grains and potentially contributed to addressing mineral deficiencies for populations with wheat-based diets, especially in As-contaminated areas. However, AMF can be potentially utilized in a conventional biofortification strategy to provide appropriate levels of minerals in grains and, thereby, help to overcome mineral deficiencies for populations with grain-based diets, especially in As-contaminated areas.

This research revealed that AMF treatment significantly decreased As(V) concentration but presented no significant effect on As(III) concentration (**Figure 3E**). Research indicates that AMF inhibited As uptake through plant roots by specifically inhibiting the high-affinity transport system, which was responsible for P uptake. This inhibition ultimately decreased As(V) absorption (Chan et al., 2013; Anawar et al., 2018). Our findings were supported by previous research studies. For example, Cattani et al. (2015) reported a significant reduction in As(V) concentration in the shoots and roots of AMF-inoculated maize, while As(III) concentration remained unaffected. Similarly, Yu et al. (2009) concluded that AMF inhibited As(V) accumulation in maize shoots, significantly lowering its uptake compared to non-mycorrhizal plants. However, its effect on As(III) uptake was not statistically significant. Indeed, AMF inoculation suppressed the activities of peroxidase, superoxide dismutase, and As(V) reductase, indicating that AMF colonization can prevent As(V) reduction to As(III). Consequently, As toxicity to plants was alleviated due to the decreased conversion of As(V) to more toxic As(III). AMF inoculation was found to play a key role in mitigating As toxicity in plants (Yu et al., 2009). In addition, AMF facilitated As methylation and volatilization, increasing the concentration of DMA and other organic substances. These findings provided valuable insights into the potential of AMF in mitigating As contamination in plants and highlighted its contribution to As detoxification processes (Li et al., 2022). Organic As toxicity is generally considered to be lower than that of inorganic As (Bali and Sidhu, 2021). Inoculation of plants with AMF could also lead to a 50.8% increase in DMA concentration (**Figure 3E**). This finding was consistent with the results obtained by Li et al. (2016), who reported a significant DMA concentration increase in rice grains following AMF inoculation. Others have reported that AMF contributed to detoxifying microbial As through processes such as methylation and volatilization (Li et al., 2018b).

### 4.2 How do soil factors affect As concentration in AMF-inoculated plants?

Although our meta-analysis revealed a significant dose-dependent AMF vaccination effect on decreasing As concentration in plants (**Figure 7A**), relatively poor data fitting the model were explained by large differences in experimental conditions (different plant species and varieties, growing periods, soil conditions, etc.) (**Figures 4**–**6**). According to our previous research findings (**Figure 4A**), soil-mediated AMF with moderate SOC levels (ranging from 0.8% to 1.5%) presented the maximum ability to decrease As concentration in plants. It is noteworthy that the amount of SOC can greatly influence AMF community structure (Qin et al., 2015). Specifically, high SOC levels increased the germination of AMF spores and mycelium and ultimately impacted AMF community composition in rhizosphere soils or roots (Luo et al., 2019). However, excessive levels of soil nutrients decreased the diversity and mycorrhizal benefits of AMF, hindering mycorrhizal symbiosis and weakening its ability to inhibit As uptake (Qin et al., 2020; Ma et al., 2021).

AMF exhibited a maximum As concentration reduction effect on plants in sandy soils or soils containing ≥50% sand, accounting for a plant As concentration reduction of 21.5% (**Figure 4A**). In contrast to sandy soils, non-sandy soils possessed greater adsorption capacities for As, and As was stabilized in the soil, which resulted in the reduction of its fluidity and solubility (Suriyagoda et al., 2018). Therefore, AMF inhibitory effect on As uptake was much more apparent in non-sandy soils. By increasing soil pH, AMF significantly reduced As concentration in host plants, which became especially pronounced under weak alkaline conditions (**Figure 4A**). As uptake by plants mainly depends on As bioavailability in soil (Kumarathilaka et al., 2018). As solubility and availability were increased as soil pH increased, leading to the release of large amounts of As in weakly alkaline soils (Yao et al., 2022). However, soil pH was the main environmental factor affecting the composition of the AMF community (Qin et al., 2015). Acidic soils inhibited AMF growth and spore germination, hindering AMF function (Liu et al., 2020). Therefore, we can conclude that the mycorrhizal effect was more pronounced in alkaline soils.

AMF exhibited the maximum reduction effect on the As concentration in plants at a medium As level. However, at higher levels of As in the soil, this inhibitory effect was weakened (**Figure 4A**). High concentrations of heavy metals and metalloids in soils can be toxic to plants, bacteria, and fungi (Parvin et al., 2019). Although AMF inoculation improved the tolerance of plants to As exposure, excessive concentrations of As in soils decreased the AMF colonization rate, negatively affecting their physiological activities and decreasing the host plant’s resistance to As symbiosis (Zhang et al., 2020). The physical and chemical properties of soils had significant effects on the AMF community, which in turn affected the germination of AMF spores and infection of hyphae, ultimately alternating As absorption and accumulation processes in plants (Tian et al., 2017; Xue et al., 2018). Strong interactions can occur between soil physicochemical properties and AMF. Therefore, further research is required to understand and optimize soil conditions under which AMF inoculation can have the most significant impact on the reduction of As concentration in plants and ecosystem protection. Meanwhile, further research is required to optimize AMF inoculation techniques, investigate genotype-specific responses, and evaluate long-term effects of AMF on As dynamics in different soil environments.

### 4.3 How do AMF inoculation methods modify As concentration in plants?

Our research revealed that several factors can influence the AMF effect on As concentration of plants. These factors included AMF inoculation species (**Figure 6A**), inoculation timing (**Figure 5A**), AMF colonization rate (**Figure 7A**), and host plants (**Figure 6B**). This was because various AMF species had distinct morphological properties, nutritional status, symbiotic efficiencies, and gene expression patterns during symbiotic interactions with plants. This research revealed a very interesting phenomenon: the interaction of AMF and crop plants affected As concentration. Maximum As reduction effects were obtained by *G. geosporum* and *R. intraradices* in Poaceae and Leguminosae (**Figure 6B**). From a food safety point of view, a significant reduction in As concentration of grains was highly desirable. This research also showed that certain varieties of Leguminosae exhibited significant suitability for AMF inoculation. Considering the significant importance of Leguminosae as a major crop plant, it was necessary to prioritize efforts aimed at decreasing As absorption and concentration in grains, specifically through AMF treatments, to improve plant health and decrease potential risks associated with As exposure.

Interestingly, the inhibitory effect of single AMF inoculation on total As levels in host plants was stronger than that of mixed AMF inoculation (**Figure 5A**). Chan et al. (2013) showed that rice grains inoculated with *G. mosseae* alone had remarkably lower total As concentrations than those inoculated with both *G. versiforme* and *G. mosseae*. Therefore, this observation suggested that fungi competed in roots and that one infection unit can prevent adjacent fungal infections, thereby avoiding secondary infections (Hepper et al., 1988; Buil et al., 2022). However, mixed inoculation presented a synergistic effect, which increased biomass and P concentration (**Figures S4**, **S6**). In fact, *Pteris vittata* co-colonized by both indigenous mycorrhizas and *G. mosseae* contained higher P concentrations than those colonized by either of the two AMFs (Leung et al., 2013).

Time plays a very important role in the biological process of AMF–plant symbiosis. Inhibitory effects of AMF on As concentration in host plants were gradually weakened with time (**Figure 5A**). This observation was consistent with the findings of Li et al. (2013). The colonization rates of the two rice cultivars exhibited significant decreases, with colonization rates ranging from 12% to 23% on day 7, from 7.3% to 11% on day 35, and from 1.3% to 4.9% on day 63. Correspondingly, shoot As concentrations appeared to be decreased on D63 when compared to D35 (*p*< 0.05) (Li et al., 2013). Initially, it was expected that longer experiments would promote the enhanced development of symbiosis, especially in situations where resources such as rooting space and nutrients were decreasing (Schroeder and Janos, 2004). However, contrary to these expectations, our findings showed that long duration levels (≥112 days) of the experiment did not result in higher mycorrhizal effects compared to short and intermediate experiments. Additionally, there was no significant difference among the three duration levels (**Figure 5A**). This is likely because As does not become limiting over time. Additionally, the results of the meta-analysis indicated a significant, dose-dependent effect of AMF infection rate on the reduction of As concentration in plants (**Figure 7A**). The duration of an experiment has a significant impact on the determining of AMF-mediated As tissue concentrations. Luo et al. (2017) found that the AMF colonization rates fluctuated with growth stages, reaching their peak at the jointing stage and then decreasing at flowering and ripening stages, but flowering and ripening stages were critical periods for AMF to limit grain Cd uptake. Wang et al. (2012) also observed a similar trend in alfalfa (*Medicago sativa* L.) that the root colonization rates by *R. intraradices* increased from 17% at day 25 to 69% at day 60 and then decreased to 43% after 80 days. This suggests that AMFs constitute an important functional component of the soil–plant system and the mechanisms cannot be explained by root colonization rates simply. Inoculation with *G. intraradices* at planting did not result in a higher root mycorrhizal colonization level than that found in non-inoculated control plants at the end of 18 months in the field. This highlights the presence of competitive processes between the natural AMF taxa and the introduced AMF strain that occurred over 18 months (Bissonnette et al., 2010). Based on the published literature, the AMF–plant symbiotic system generally exhibits ecological functions throughout the entire life span of a plant. Meanwhile, different symbiotic systems have varying time periods during which they exhibit maximum functionality. As the plant’s life cycle comes to an end, the colonization rate of AMF significantly decreases. However, it should be noted that the time period during which AMF exerts maximum functionality does not necessarily coincide with the time of maximum colonization rate.

Although we have obtained the above general conclusion, we must acknowledge that the colonization rate alone cannot adequately explain the mechanism of AMF tolerance to As. Feddermann et al. (2010) suggested that AMF inoculated with different host plants may possess different nutritional status, symbiotic efficiency, and gene expression patterns leading to distinct colonization rates. Liu et al. (2005) found that the shoot As concentration of rice with AMF inoculation decreased at lower rates of As application to the soil (<50 mg/kg), with the opposite trend at higher soil As levels. There was a break point at the added As level of 50 mg/kg at which the lowest proportion of As was distributed in the shoots (Liu et al., 2005). Long et al. (2021) also found that when adding 10 g/kg iron tailings (IT), AMF significantly increased root As concentration (*p*< 0.05), while at 40 g/kg iron tailings (IT), AMF decreased As concentration in shoots by reducing As absorption efficiency. This suggests that soil arsenic levels also influence AMF-mediated uptake and translocation of As in host plants. In fact, the effect of As concentration in plants mediated by AMF symbiosis was influenced by many factors, including plant species (AbdElgawad et al., 2022), plant growth stage (Chen et al., 2006), AMF species (Bhalla and Garg, 2021), physical and chemical properties of soil (Coutinho et al., 2015), nutritional status (Hajiboland et al., 2009), and soil amendment Bissonnette. Our findings also supported this viewpoint (**Figures 4**–**6**). The relatively poor fits of the data to the model were explained by the large difference in experimental conditions (different plant species and varieties, AMF species, growing period, soils, etc.) of the 76 studies that matched the selection criteria (**Figure 7A**). The contradictions in the literature regarding the effects of AMF on plant As response can be attributed to the complex soil–AMF–plant mechanisms involved, which cannot be solely evaluated based on root colonization rates.

### 4.4 How do P fertilizers and AMF modify As concentration in plants?

Our meta-analysis presented compelling quantitative evidence demonstrating a significant decrease in As concentration with the increase of the P/As ratio in the plant. In addition, a significant negative correlation (*p*< 0.05) was observed between the P/As ratio and As concentration in total plants and grains (**Figures 8C, D**). This finding was important since P competed with As for plant uptake, limiting As absorption and translocation in plants (Li et al., 2016). The symbiotic relationship between AMF and plants can effectively reduce As influx into roots and enhance the accumulation of plant nutrients, resulting in a “dilution effect” on As in plant tissues. Consequently, As concentration in crops was effectively decreased, mitigating the risk of As exposure to humans through dietary intake (Cheema and Garg, 2022). In addition, AMF facilitated As translocation from the host plants to their hyphae, enabling the fungi to effectively remove As from plant systems and prevent its accumulation in host tissues. This translocation process further helped to decrease As concentration within plants (Li et al., 2018a). However, under low and medium P supply conditions (P levels of below 60 mg/kg), AMF inoculation did not significantly affect As levels in plants (**Figure 5A**). This finding was interesting because it suggests that the AMF effect on decreasing As concentration in plants depended on the availability of P in soil. In other words, when P was limited, AMF might be unable to fully exert its potential in decreasing As accumulation. However, under high P conditions (P levels equal to or above 60 mg/kg), AMF inoculation was found to significantly decrease As concentration in plants (**Figure 5A**). This result showed that when plants had sufficient P supply, AMF could effectively increase the uptake and utilization of P, improving plant growth and diluting As accumulation. This finding highlighted the importance of considering P availability in soil when implementing AMF as a strategy for mitigating As contamination. Optimizing soil P levels through fertilization or soil amendment can potentially improve AMF effectiveness in decreasing As accumulation in plants.

AMF inoculation alleviated As toxicity to plants through two mechanisms. First, it upregulated the levels of low-affinity P transporters, thereby improving P absorption efficiency and assisting host plants in acquiring more P (Paszkowski et al., 2002). Second, it downregulated the expression levels of high-affinity P transporters on the root surface and hair, decreasing As uptake (Gupta et al., 2022). Both of the above mechanisms contributed to As toxicity mitigation in plants. Overall, the shoot P/As ratio served as a valuable indicator for evaluating the beneficial effects of AMF species in enhancing As tolerance in various plant species (Mitra et al., 2022). In fact, the shoot P/As ratio was found to be a critical indicator for investigating As concentrations in both plants and grains (**Figures 8C, D**). In addition, the shoot P/As ratio can help identify plants or varieties exhibiting higher As tolerance or lower As accumulation, assisting in breeding programs and agricultural practices aimed at minimizing As uptake in crops.

### 4.5 What are the general mechanisms of AMF regulation on the bioavailability and accumulation of As in plants, as proposed in literature and presented data?

This research showed that inoculating AMF significantly enhanced the concentrations of EE-GRSP and T-GRSP in As-contamination soils (**Figure 9**), which was consistent with the findings of previous studies (Li et al., 2018a; Zhang et al., 2020). GRSP, a glycoprotein produced by AMF in soil, has been extensively recognized for its ability to bind to toxic metals, thus contributing to metal stabilization (Chen et al., 2022). Previous research studies have revealed a significant correlation between GRSP and mycorrhizal root volume (Bedini et al., 2009). Others have observed that higher glomalin contents corresponded to a greater expansion of AMF extraradical hyphae (Zhang, H. et al., 2016). This observation suggested that increased GRSP contents might contribute to longer AMF extraradical hyphae and faster turnover of those hyphae compared to no-inoculation treatment (Zhang et al., 2020). In fact, this consequence explained the potential reasons why AMF inoculation enhanced plant tolerance to toxic As in soils. However, further research is still required to determine whether glomalin can directly bind to As, decrease As bioavailability in soil, and subsequently decrease As uptake by plants.

Finally, by integrating previous analysis results and literature, we outlined a conceptual flow diagram depicting the role of AMF in regulating the bioavailability and accumulation of As in plants (**Figure 10**). **Figure 10** shows the potential of AMF–plant symbiosis systems to enhance dry biomass, increase P concentration, and subsequently decrease As concentration in plants. Tolerance of host plants to toxic As mainly relies on As uptake reduction in AMF plants, which is accomplished through different mechanisms, including the following.

1) Downregulation of As transporters: As(III) is taken up by plants through the silicon transporter (Lsi1), and As(V) is taken up and transported by phosphate transporters (PhTs) (Ma et al., 2008; Zhao et al., 2009). Inoculation with AMF led to the downregulation of *Lsi1* and *Lsi2* in rice roots, resulting in reduced As(III) uptake and transport (Chen et al., 2012). In rice, inoculation with AMF downregulated the expression of several *OsPT* genes (*OsPT1–3*, *OsPT6*, and *OsPT9–10*) (Chen et al., 2013).
2) Upregulation of P transporters: Inoculation with AMF significantly enhanced P nutrition in plants and limited As uptake through the upregulation of the AM-induced *PhT* gene, *MsPT4* (Li et al., 2018a). Additionally, the activity of *Pht1;5* and *Pht1;6* increased and exhibited higher selectivity toward P than As, effectively reducing the absorption of As in AMF symbiosis (Christophersen et al., 2012).
3) Sequestration, methylation, and volatilization of As: Mycorrhizal association can lead to involvement in sequestration (storage), deposition (in external mycelium), chelation (form stable metal–organic complexes), excretion [As(III) excretion to external media], methylation (conversion to less toxic forms), and volatilization (release into the air) of As (Liu et al., 2005; Li et al., 2016; Li et al., 2018a, b; Xing et al., 2024). These processes helped in decreasing the overall As load in plants.
4) Improved physiological function: AMF can mitigate the oxidative stress induced by As, enhance the activity of the antioxidant enzyme system, and improve photosynthesis. By reducing the production of hydrogen peroxide (H_2_O_2_) and lipid peroxidation, AMF effectively countered oxidative damage in host plants (Shukla et al., 2023; Zhou et al., 2023). This symbiotic relationship significantly increased the activity of essential antioxidant enzymes, including catalase (CAT), peroxidase (POD), and superoxide dismutase (SOD) (Cheema and Garg, 2022, 2024; Mitra et al., 2022). AMF facilitated the restoration of pigment levels, including chlorophyll *a*, chlorophyll *b*, and carotenoids, and enhanced photosynthetic efficiency, transpiration rates, and water use efficiency under conditions of As stress (Zhang et al., 2022; Shukla et al., 2023).
5) Increased the accumulation of nutrients: AMF not only facilitated the enhanced uptake of vital macronutrients but also significantly boosted the accumulation of critical nutrients, including nitrogen (N), P, potassium (K), calcium (Ca), and magnesium (Mg), in the plant grains. By increasing the host plants’ biomass, AMF also contributed to a reduction in the As concentration through a dilution effect, effectively mitigating the toxic effects of As exposure (Gupta et al., 2022).

To obtain a comprehensive understanding of the mechanisms applied by AMF to improve As tolerance in plants, further biochemical and physiological characterization is necessary. This knowledge will guide strategies to improve plant resilience to As contamination and contribute to developing sustainable and effective solutions for the management of As in agricultural systems.

## 5 Conclusions and prospects

Meta-analysis of 1,362 data records retrieved from 76 published studies evaluated the effects of various abiotic and biotic factors on AMF-related As accumulation in plants by subgroup and regression analyses. The findings of this research were as follows: AMF infection significantly lowered plant As levels in a dose-dependent manner (*p*< 0.05) and enhanced total P concentration and plant dry weight. Notably, As(V) levels decreased and DMA levels increased with AMF inoculation, but As(III) levels were unaffected. Single AMF inoculation was more effective at reducing As in plants than mixed inoculation. The optimal soil conditions for AMF are a SOC range of 0.8%–1.5% and a pH of ≥7.5 for maximum As reduction in plants. AMF inoculation increased the P/As ratio in plants, with a significant negative correlation (*p*< 0.05) between this ratio and As grain concentration. Among common AMF species, *R. intraradices* and *F. mosseae* were effective in reducing As in plants, with *R. intraradices* showing particular promise in Leguminosae plants, making them strong candidates for further research. AMF also increased EE-GRSP and T-GRSP concentrations in As-contaminated soils, offering a potential strategy to reduce As exposure and intake through the human diet, thereby enhancing human health.

For a further understanding of how AMF affects As accumulation in plants, future research should focus on 1) conducting field trials to optimize AMF use in biofortification, considering crop–AMF interactions, inoculation methods, soil properties, agricultural practices, and AMF’s long-term impact on As dynamics; and 2) investigating the mechanisms of AMF–plant synergy in As tolerance, including variability in AMF efficacy, environmental interactions, underlying metabolic pathways or signaling molecules, and key enzymes or proteins. Further in-depth research is needed to comprehensively understand the mechanisms by which AMF improves the As tolerance of plants.

## Author contributions

SH: Data curation, Software, Visualization, Writing – original draft. YT: Data curation, Software, Visualization, Writing – original draft. ZL: Writing – review & editing. LX: Data curation, Software, Writing – original draft. XZ: Conceptualization, Visualization, Writing – review & editing. GB: Writing – review & editing.
