# Supplementary material for: Effects of arbuscular mycorrhizal fungi on the reduction of arsenic accumulation in plants: a meta-analysis
Source: Front Plant Sci. 2024 Apr 5;15:1327649. doi: 10.3389/fpls.2024.1327649 (PMC11026667; doi:10.3389/fpls.2024.1327649)
Supplement: Supplementary file 1 [file DataSheet_1.docx]

Supplementary Material

# Supplementary Figures and Tables

## Supplementary Figures


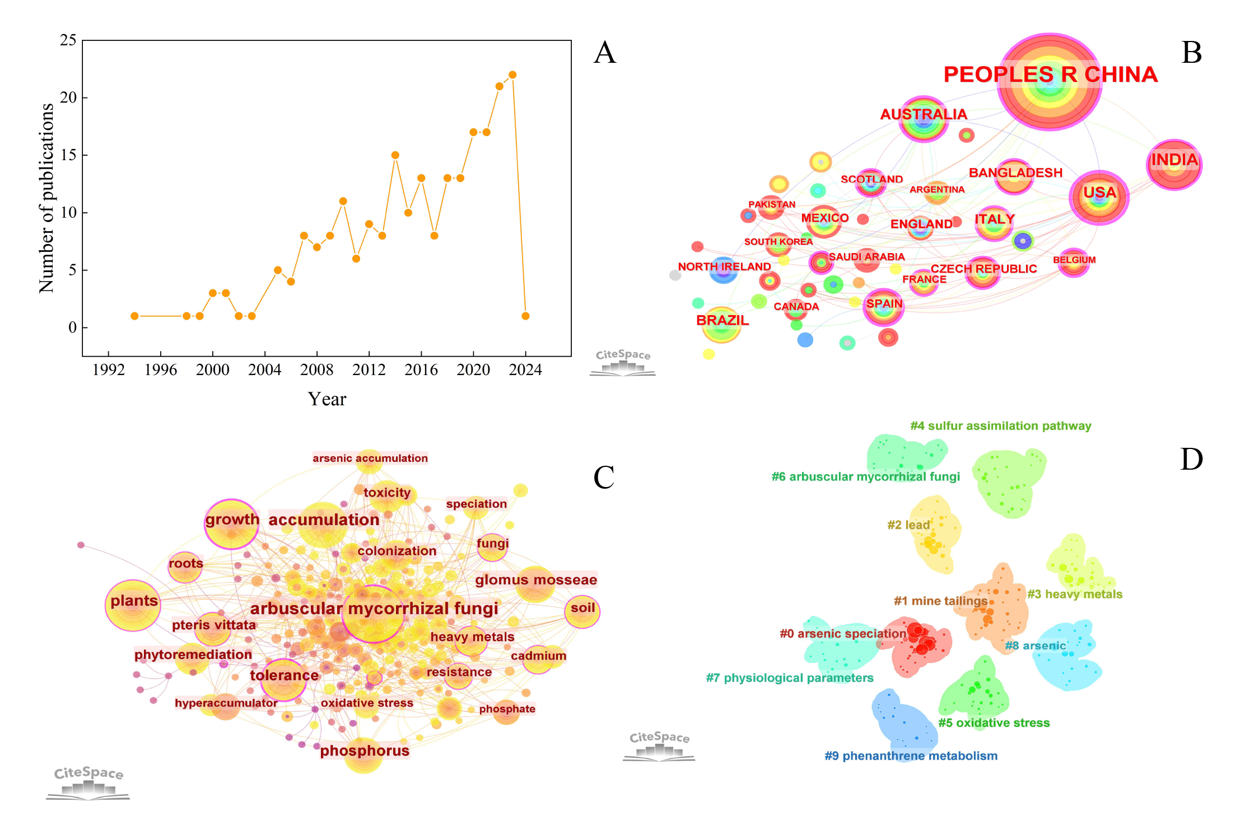
**Supplementary Figure 1.** Visualization diagrams of CiteSpace. Number of annual publications (A). The cooperative relationship map of the countries (B). Relative engagement of countries conducting research on AMF for remediating As pollution. Node size signifies the number of papers that originated from the country, the distance between nodes and link thickness indicates the level of collaboration. Visualization of keyword co-occurrence network (C). Each node represents a keyword, and the larger the node, the more frequently the category appears. Cluster of keyword co-occurrence map (D). The colors represent different clusters.


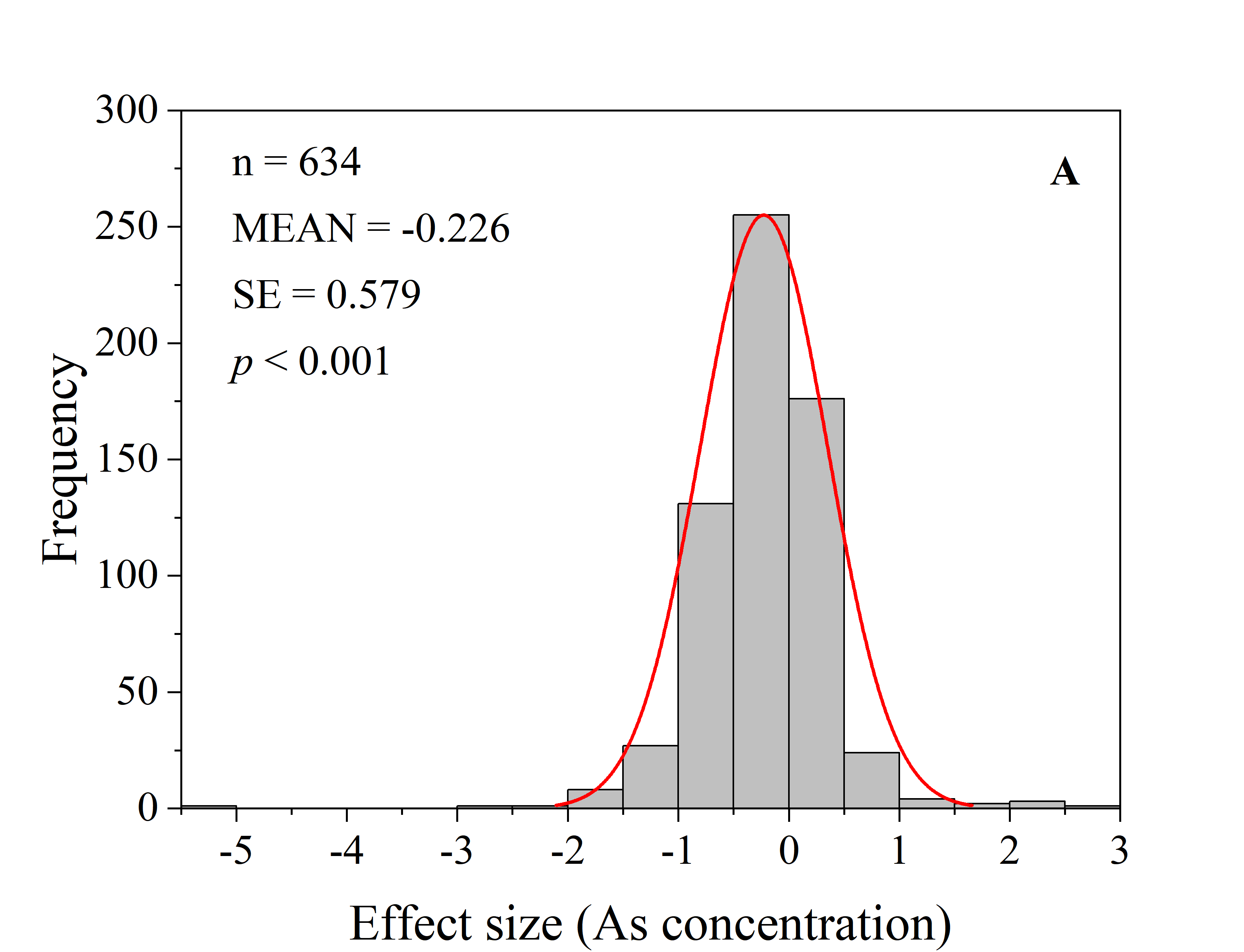

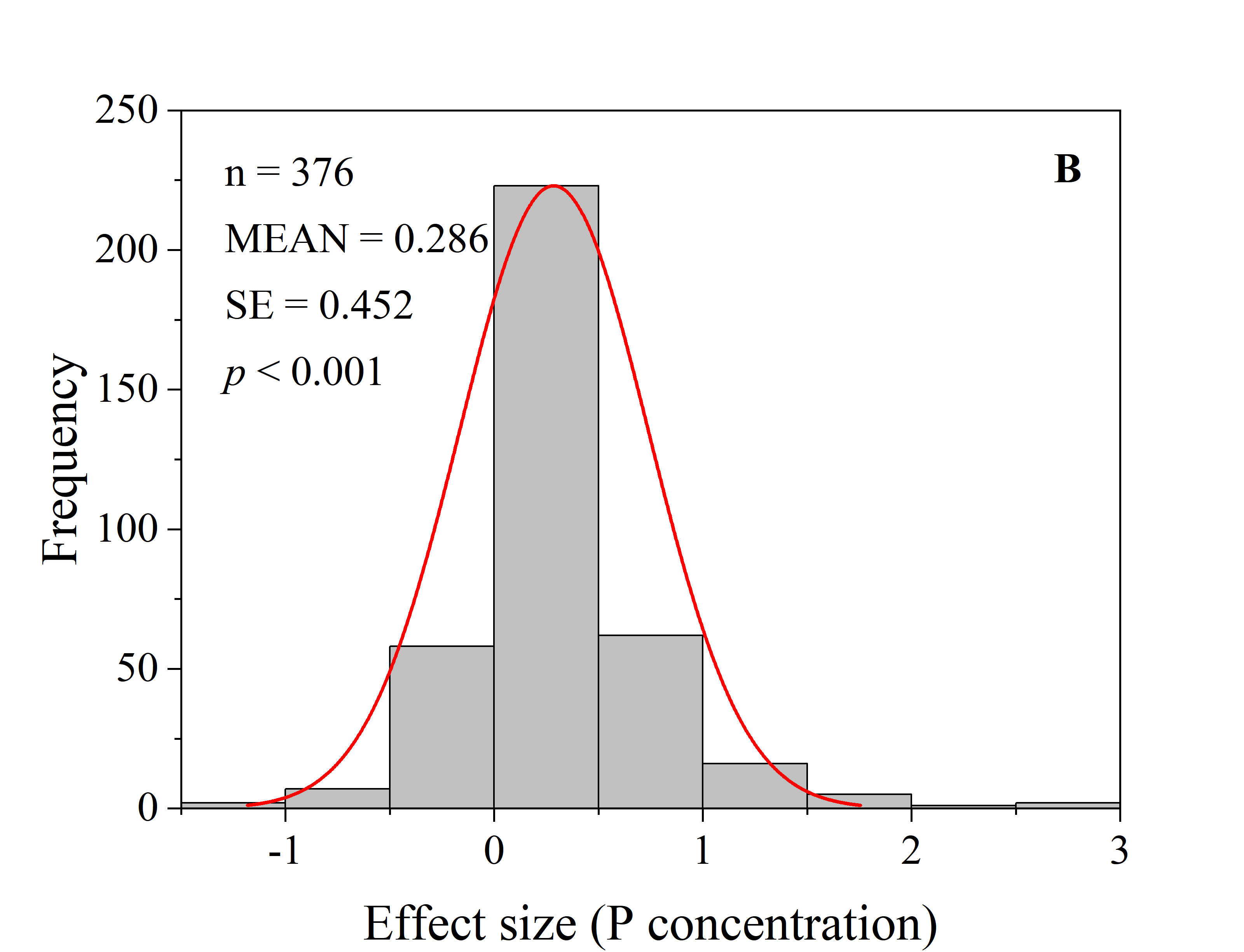

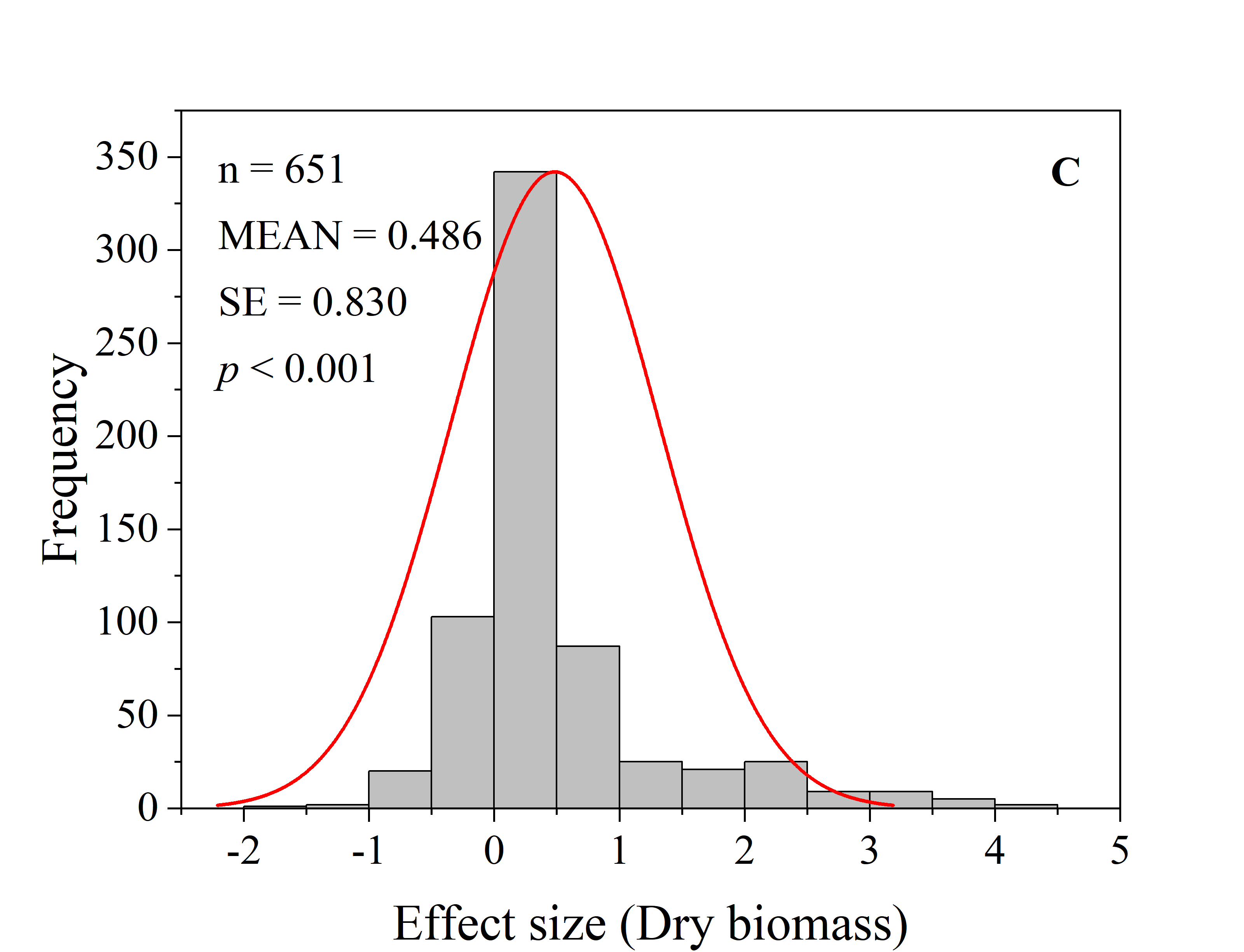


**Supplementary Figure 2.** Effect size frequency distribution histogram of As concentration (A), P concentration (B) and dry biomass.


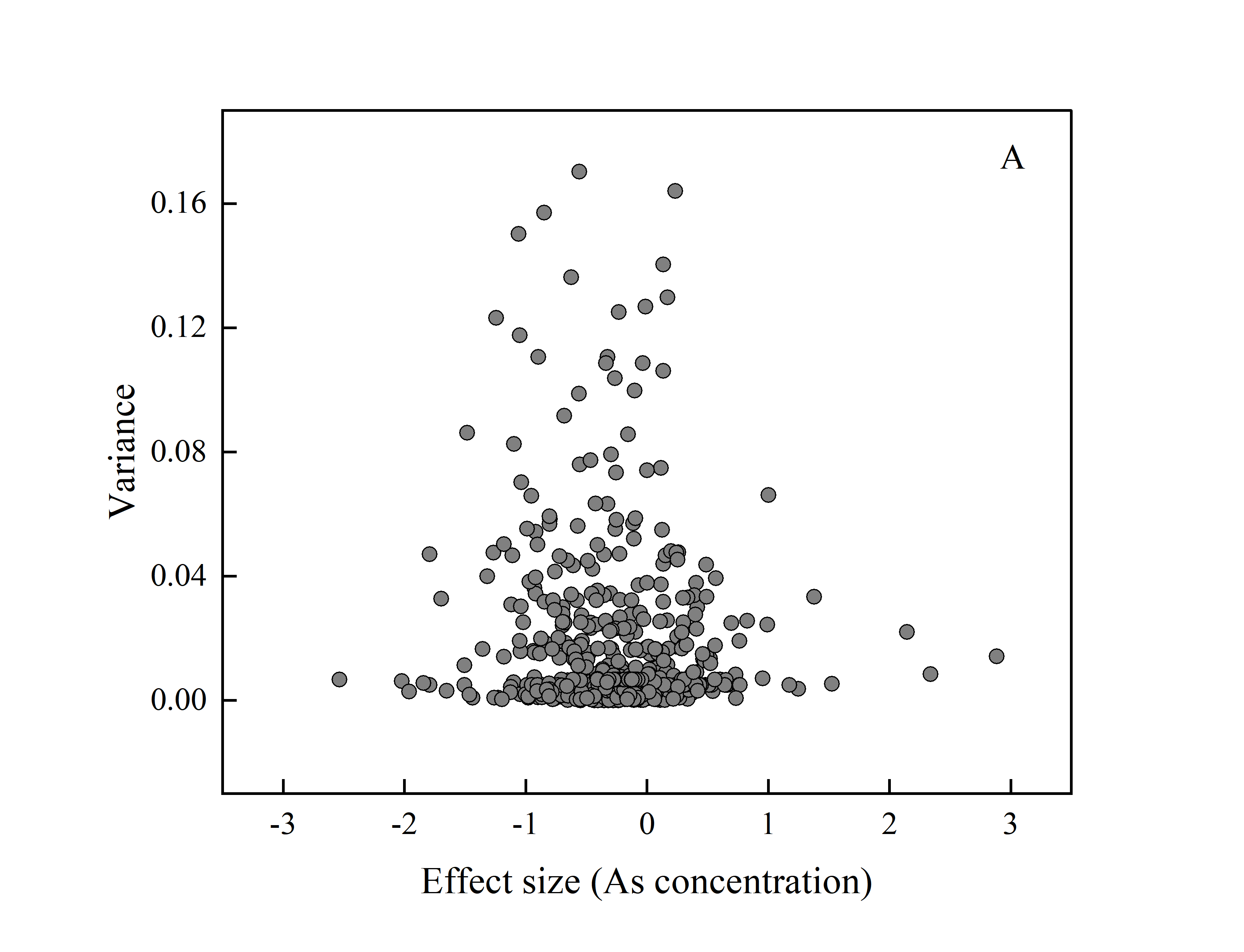

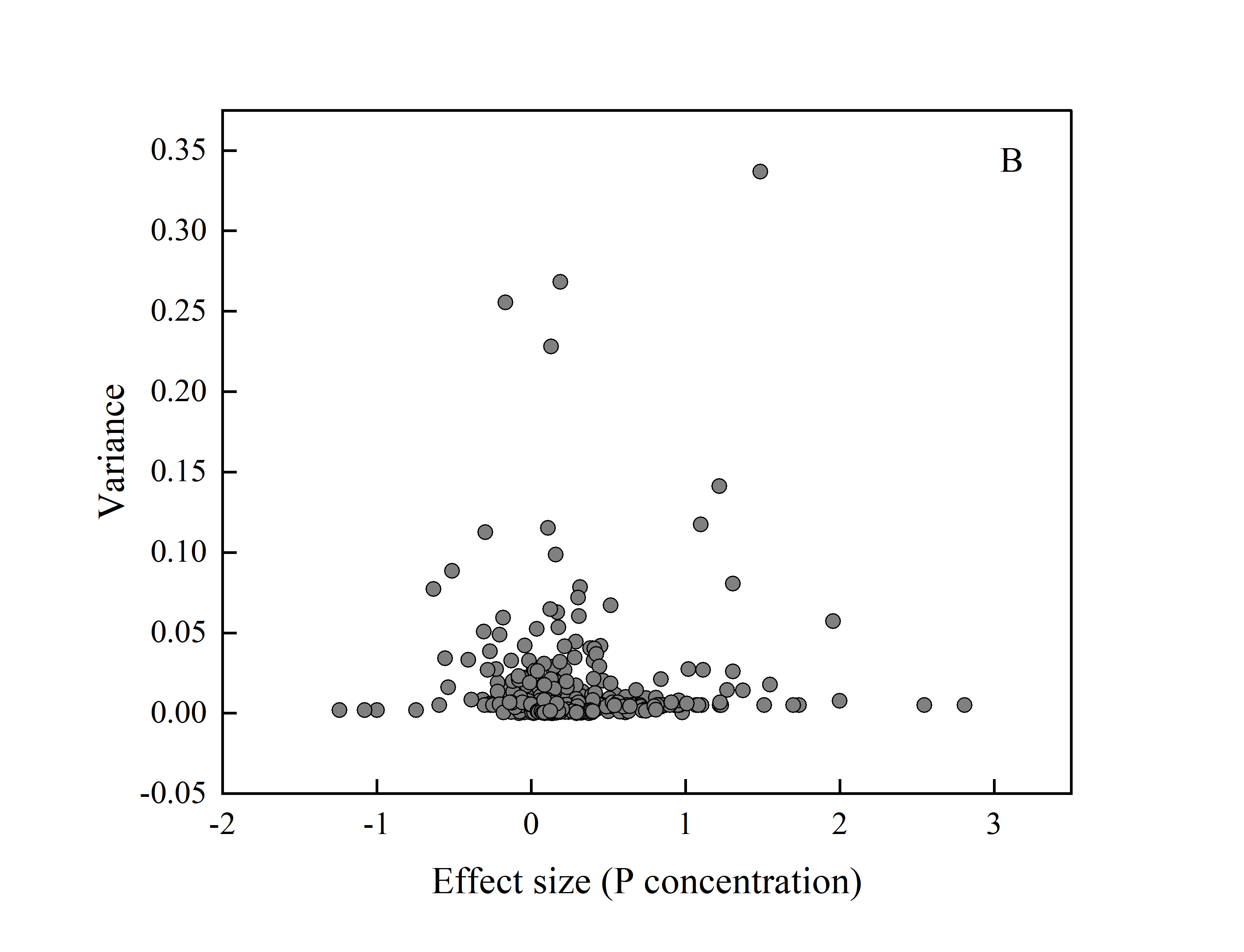

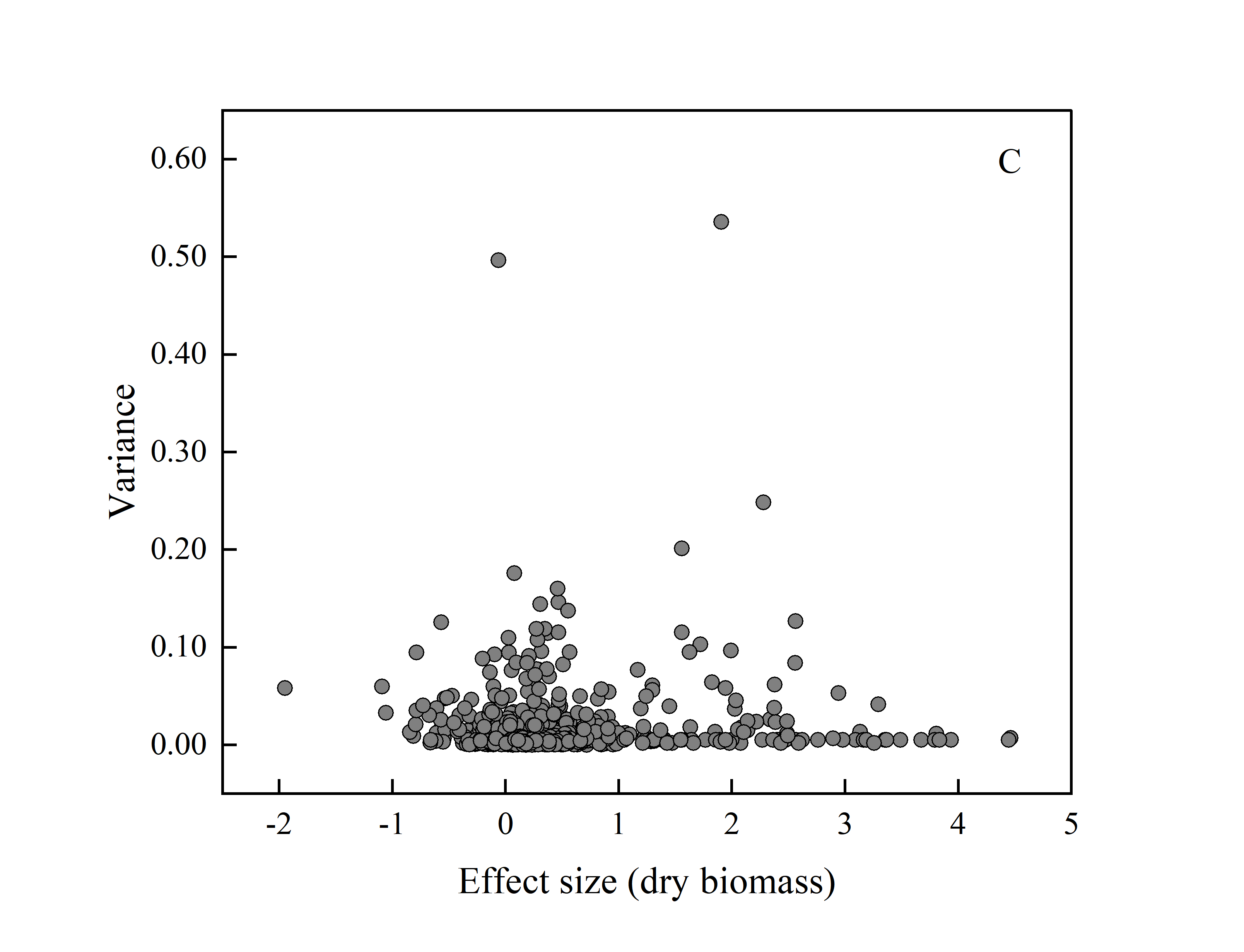


**Supplementary Figure 3.** Funnel plot of As concentration (A), P concentration (B) and dry biomass (C).

**Supplementary Figure 4.** Effects of different soil properties on P concentration in total plants (A), grains (B), shoots (C) and roots (D) with AMF inoculation.


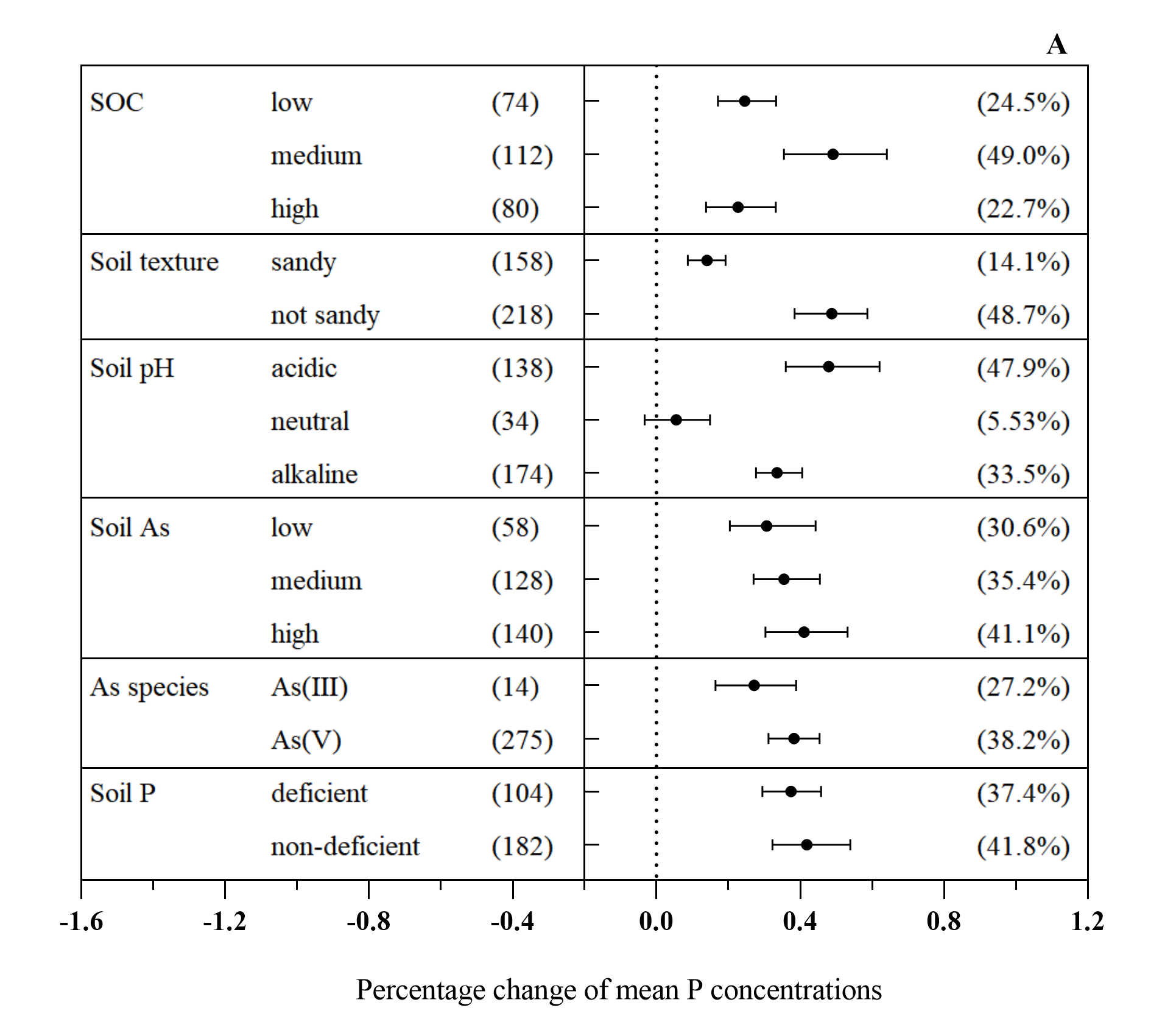

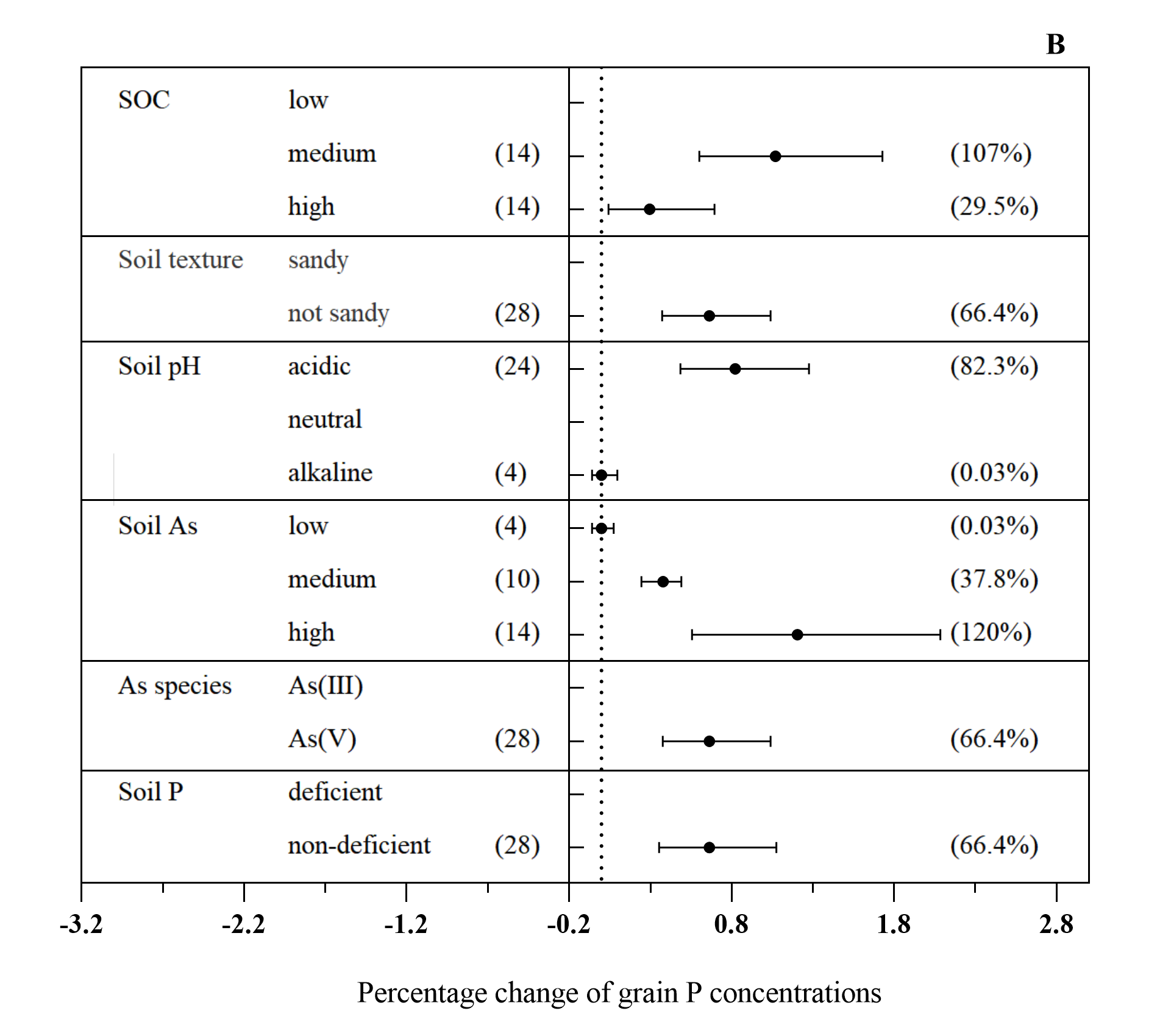

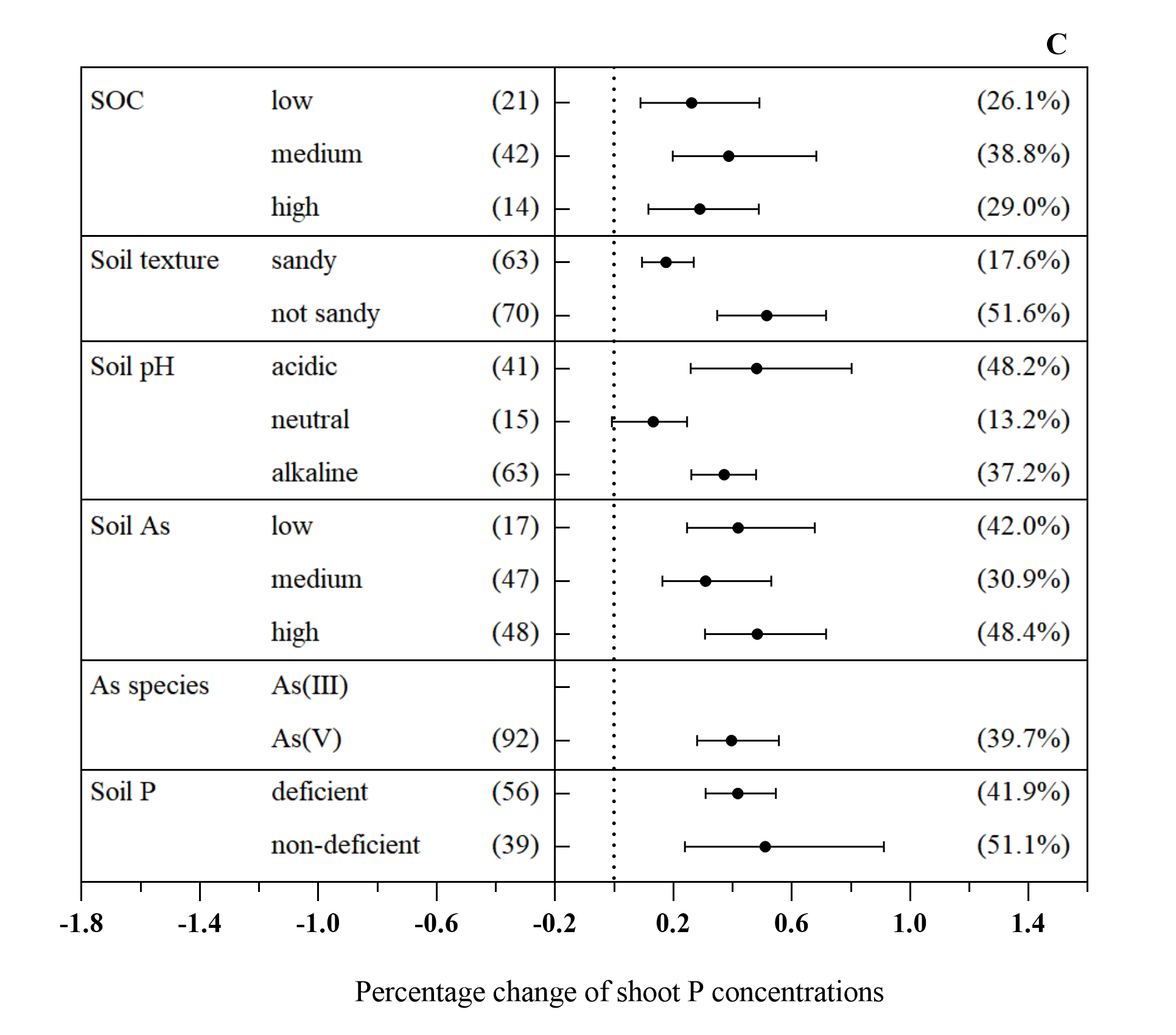

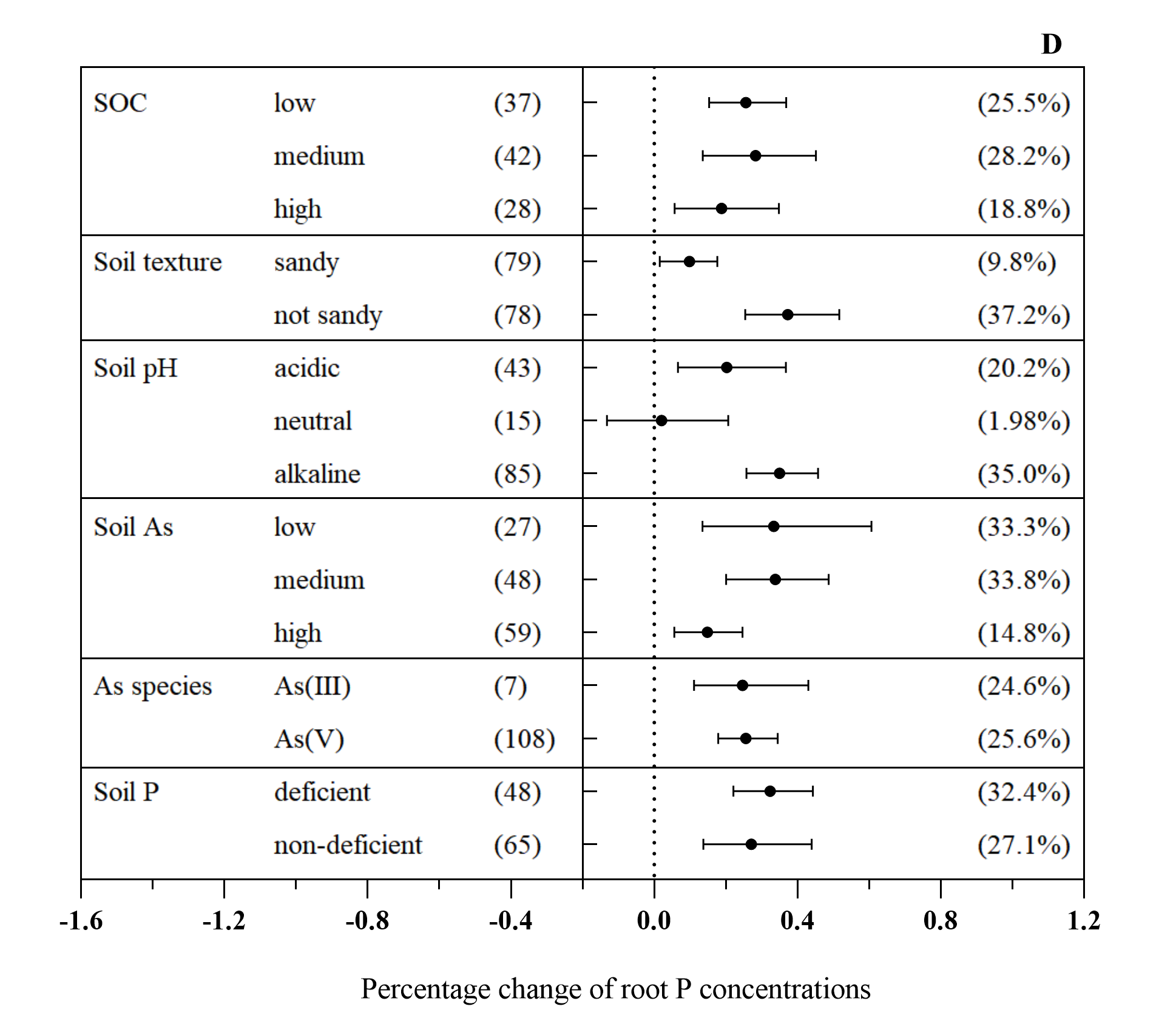


**Supplementary Figure 5.** Effects of different external factors on P concentration in total plants (A), grains (B), shoots (C) and roots (D) with AMF inoculation.


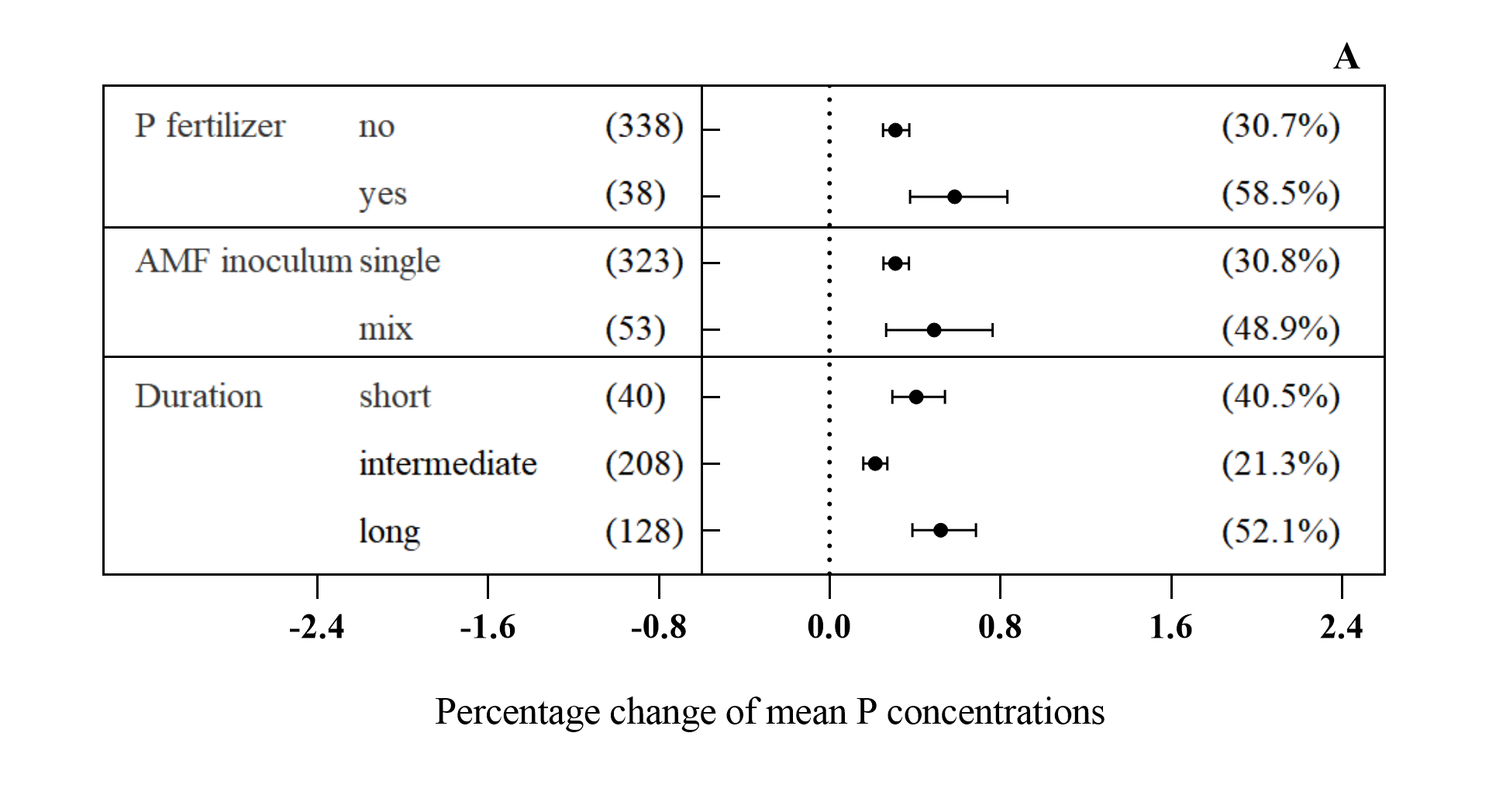

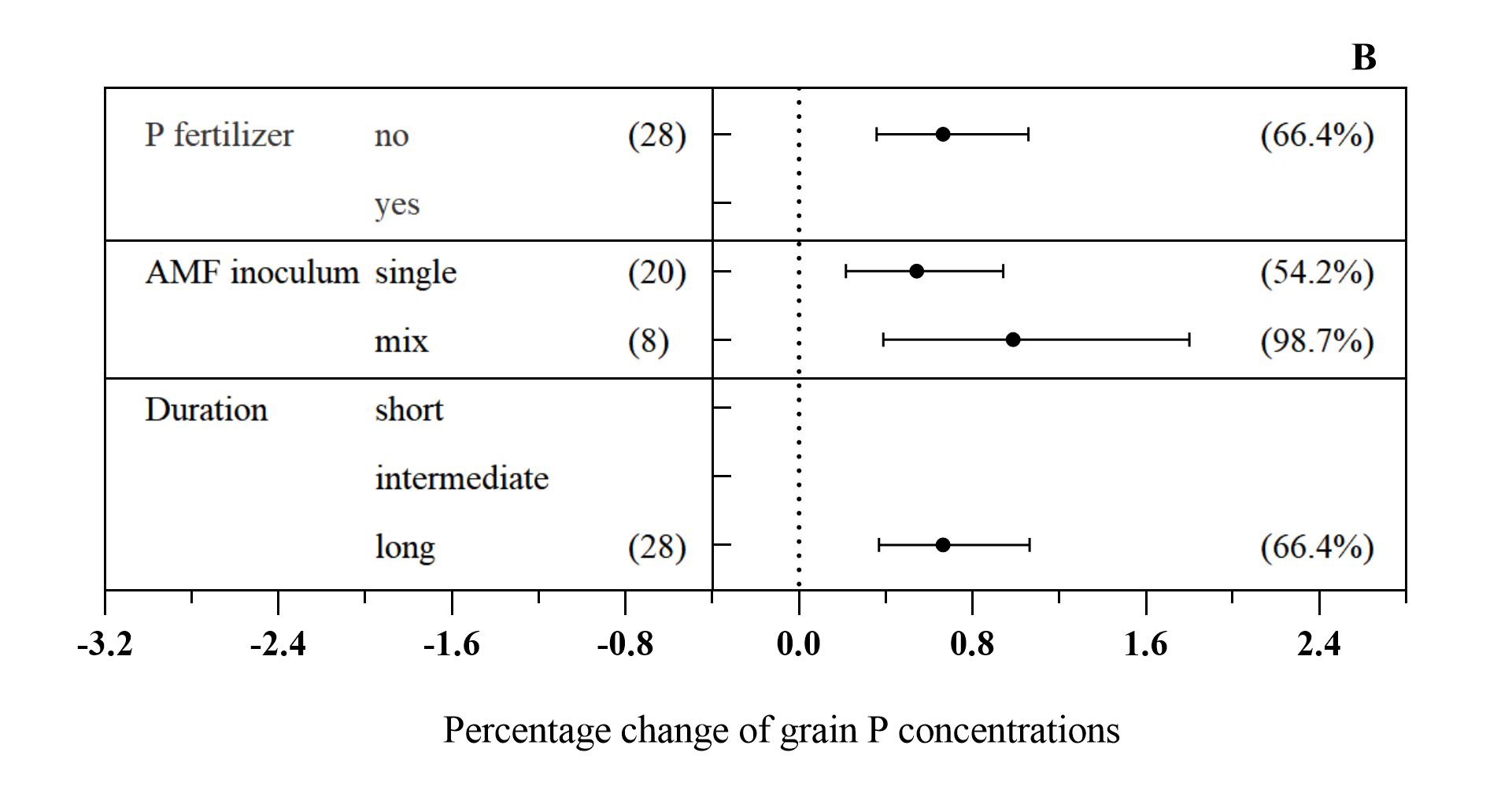

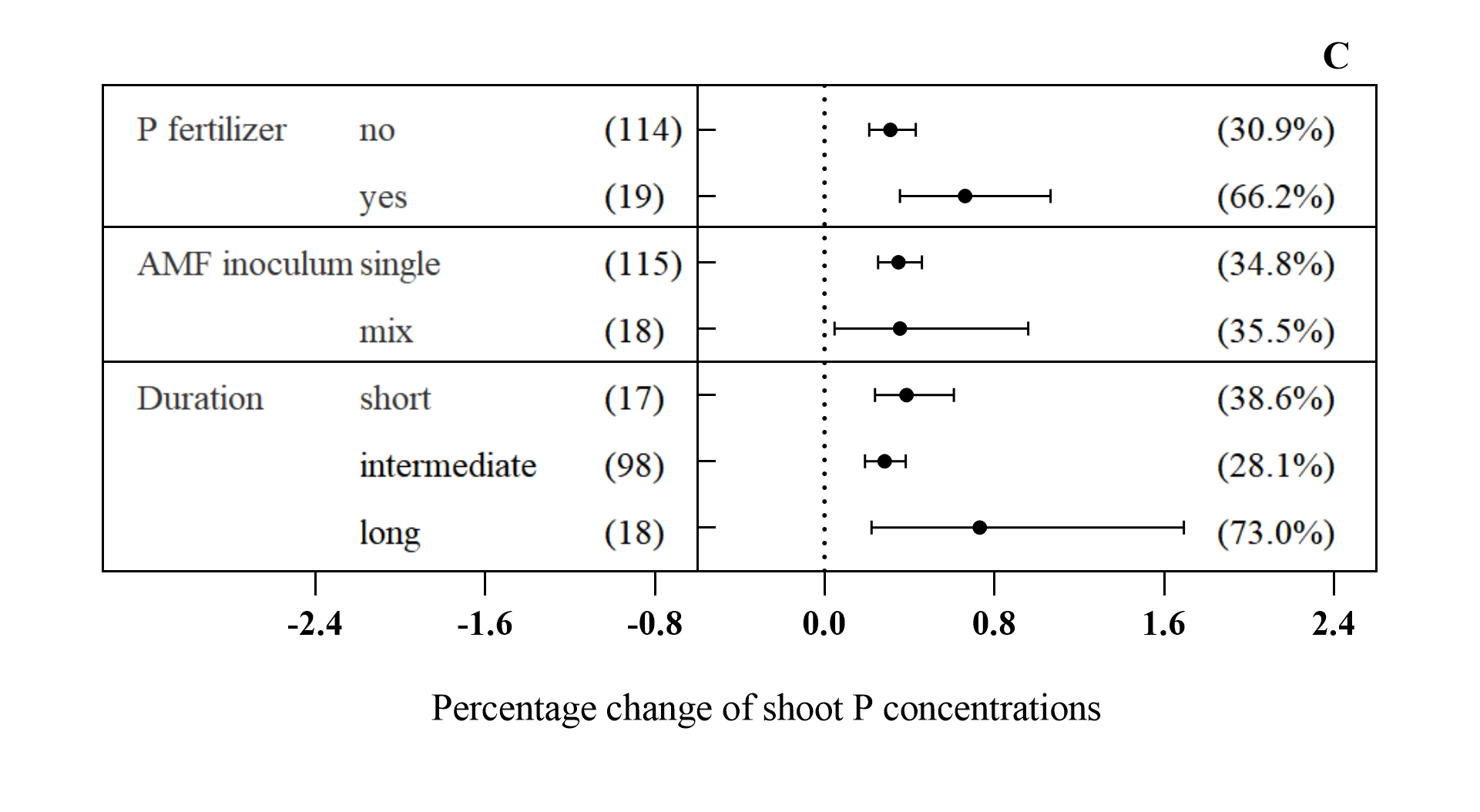

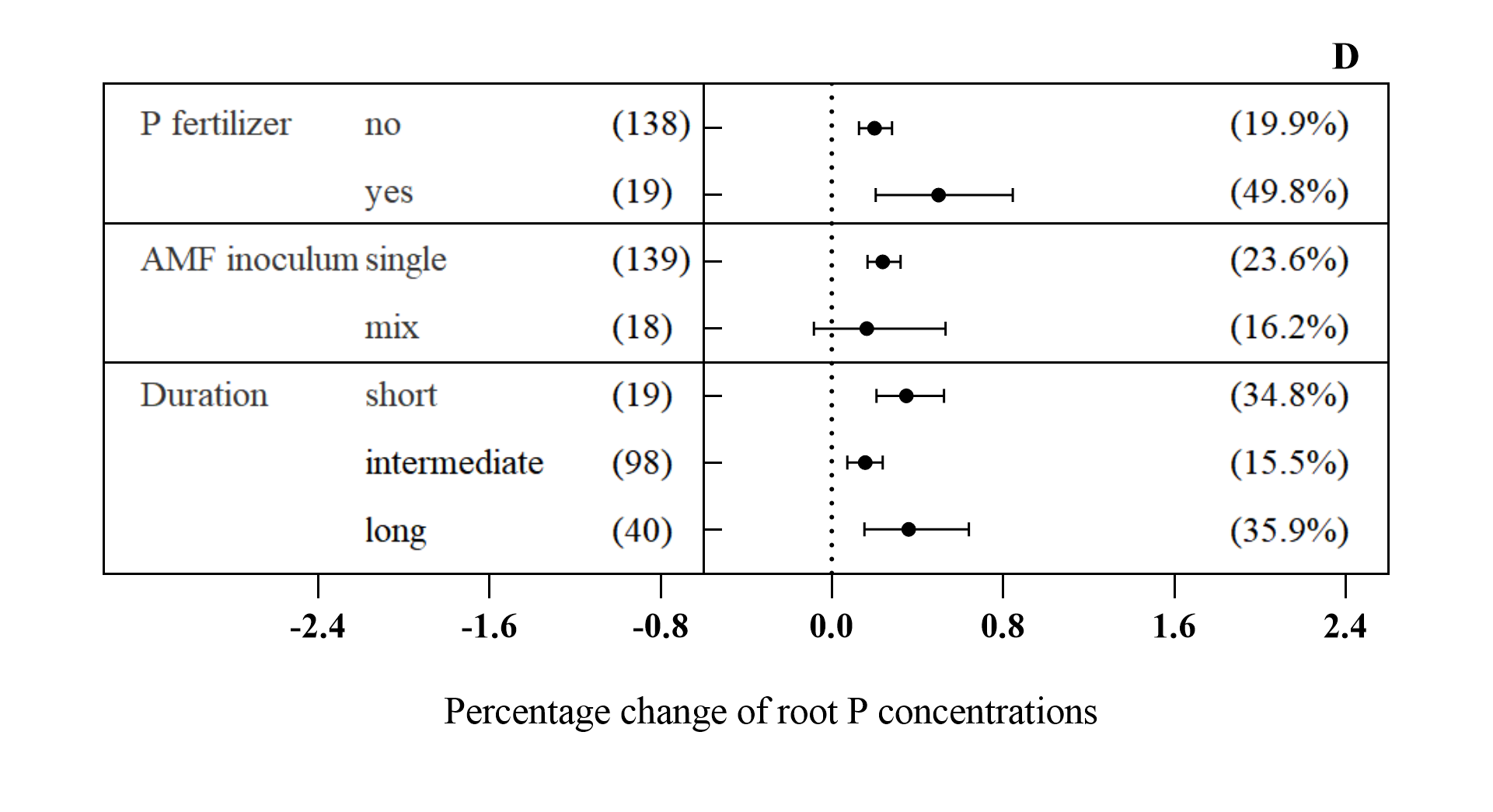


**Supplementary Figure 6.** Effects of different soil properties on dry biomass in total plants (A), grains (B), shoots (C) and roots (D) with AMF inoculation. **Supplementary Figure 7.** Effects of different external factors on dry biomass in total plants (A), grains (B), shoots (C) and roots (D) with AMF inoculation.


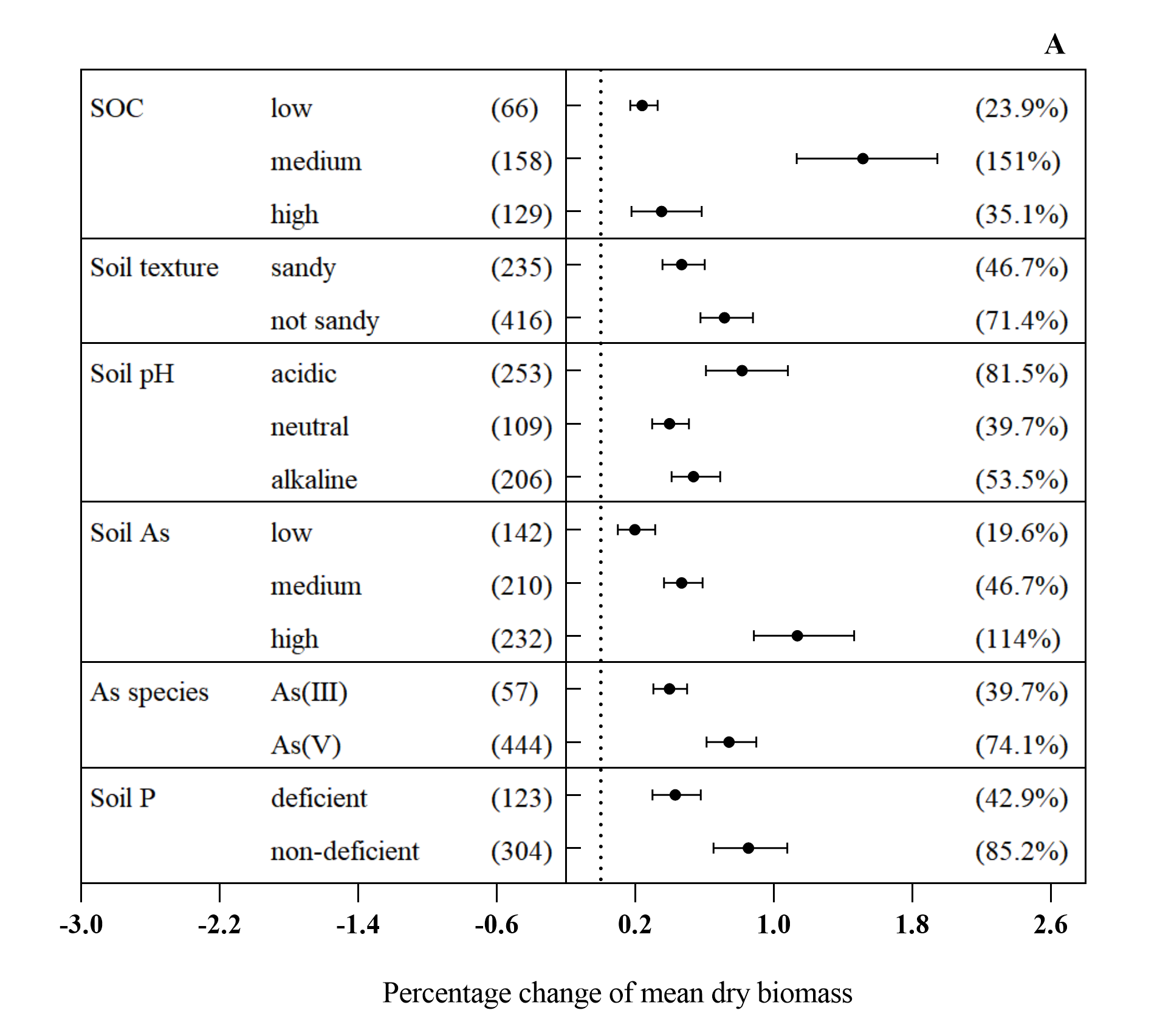

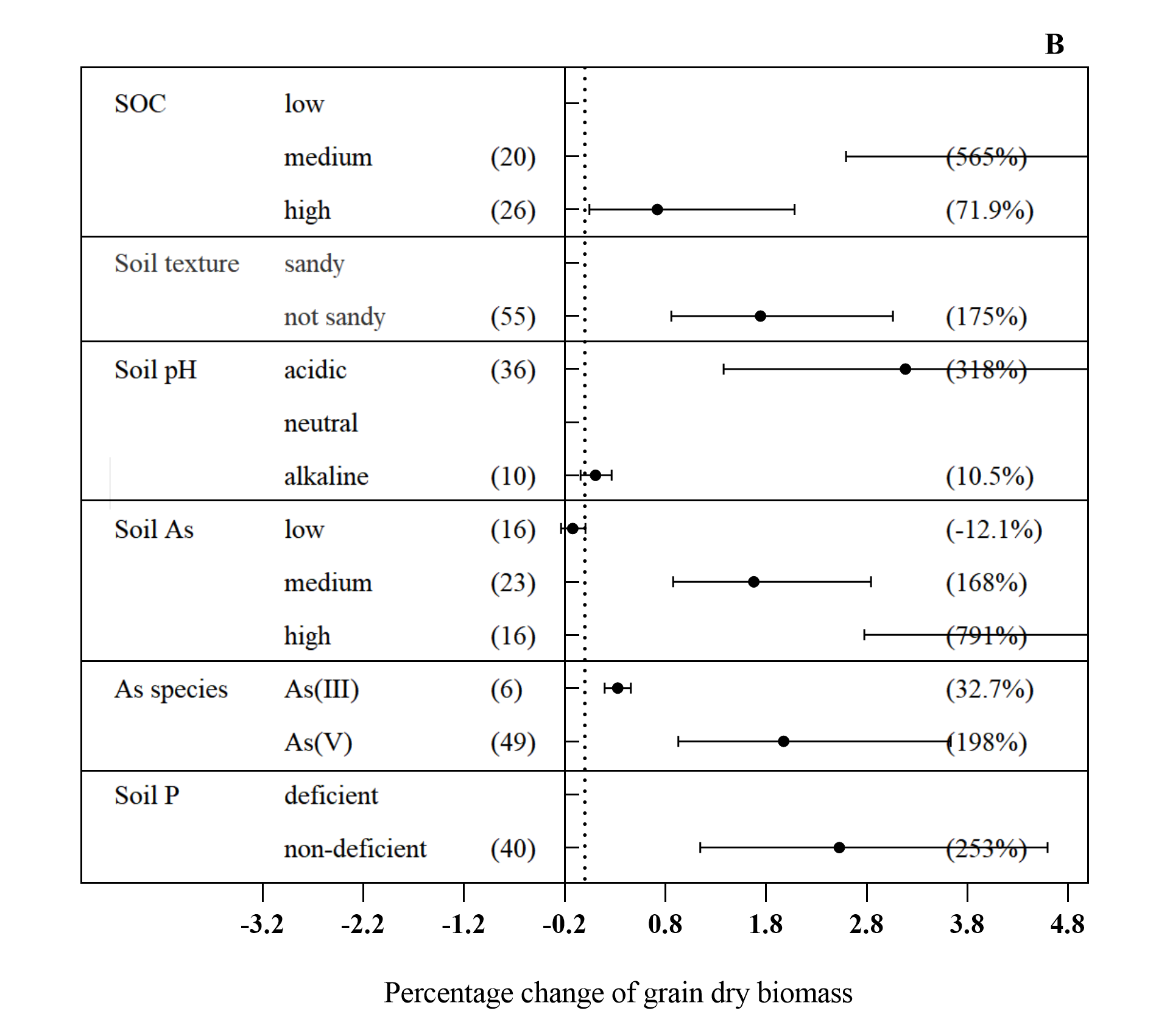

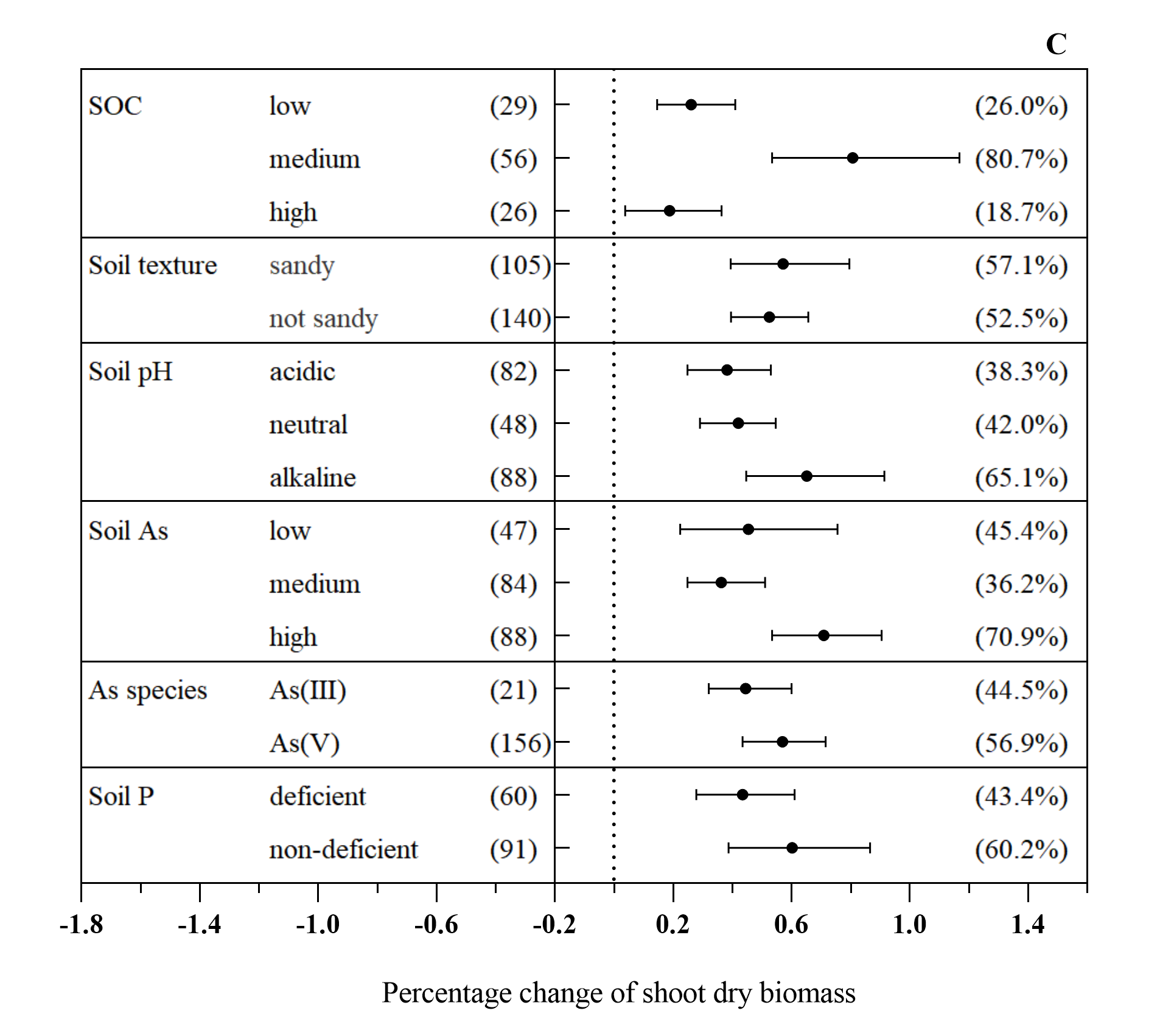

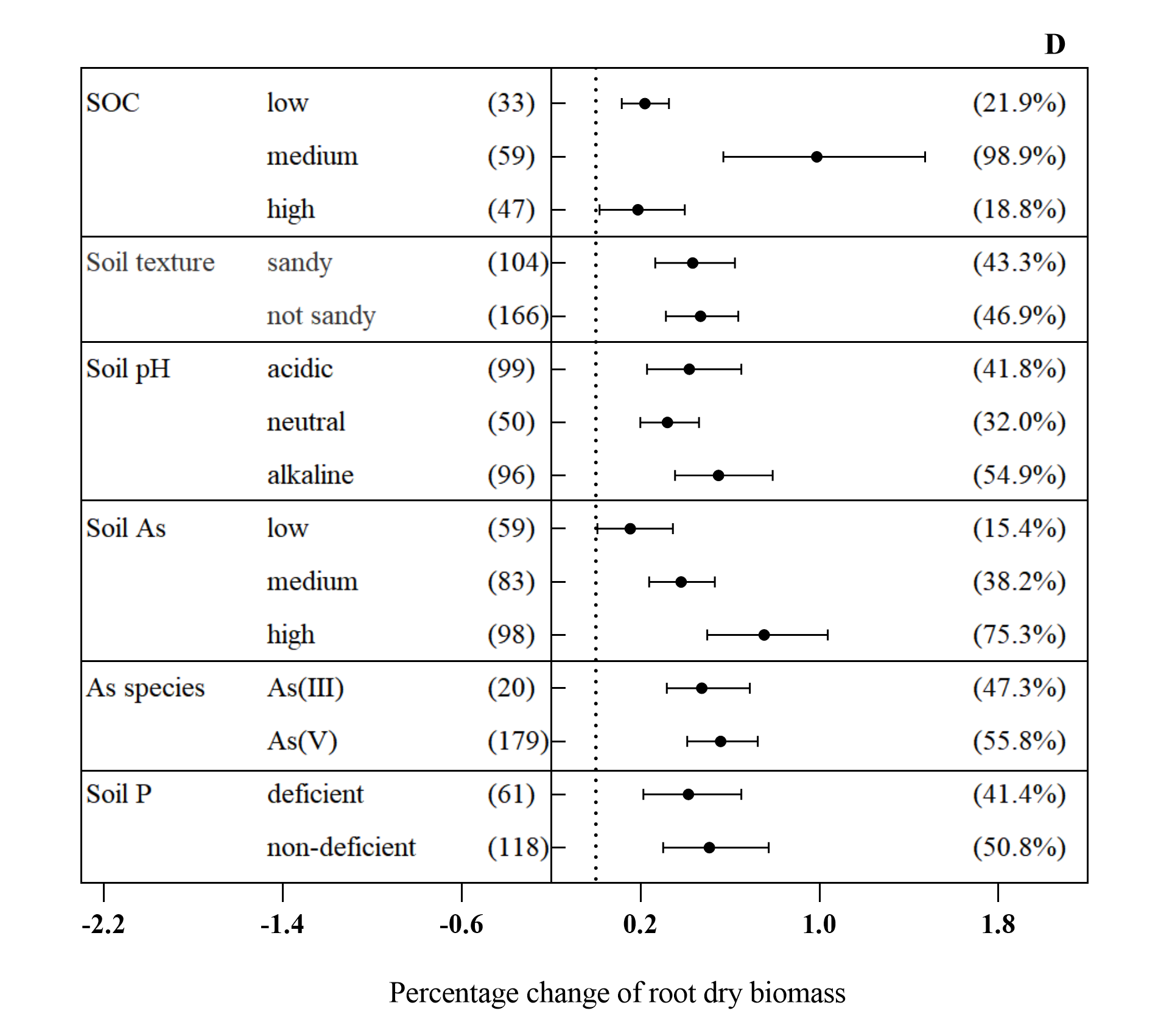

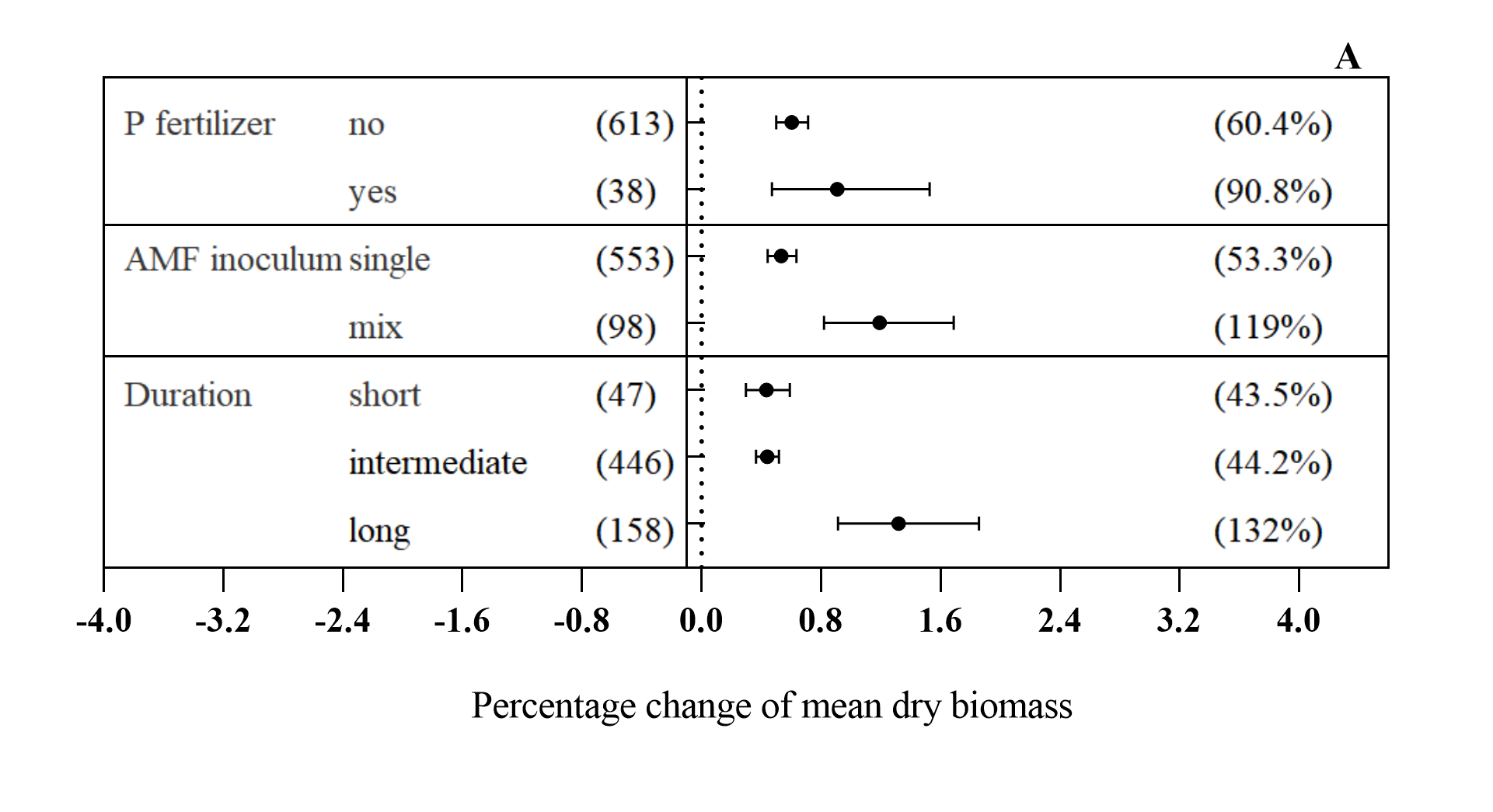

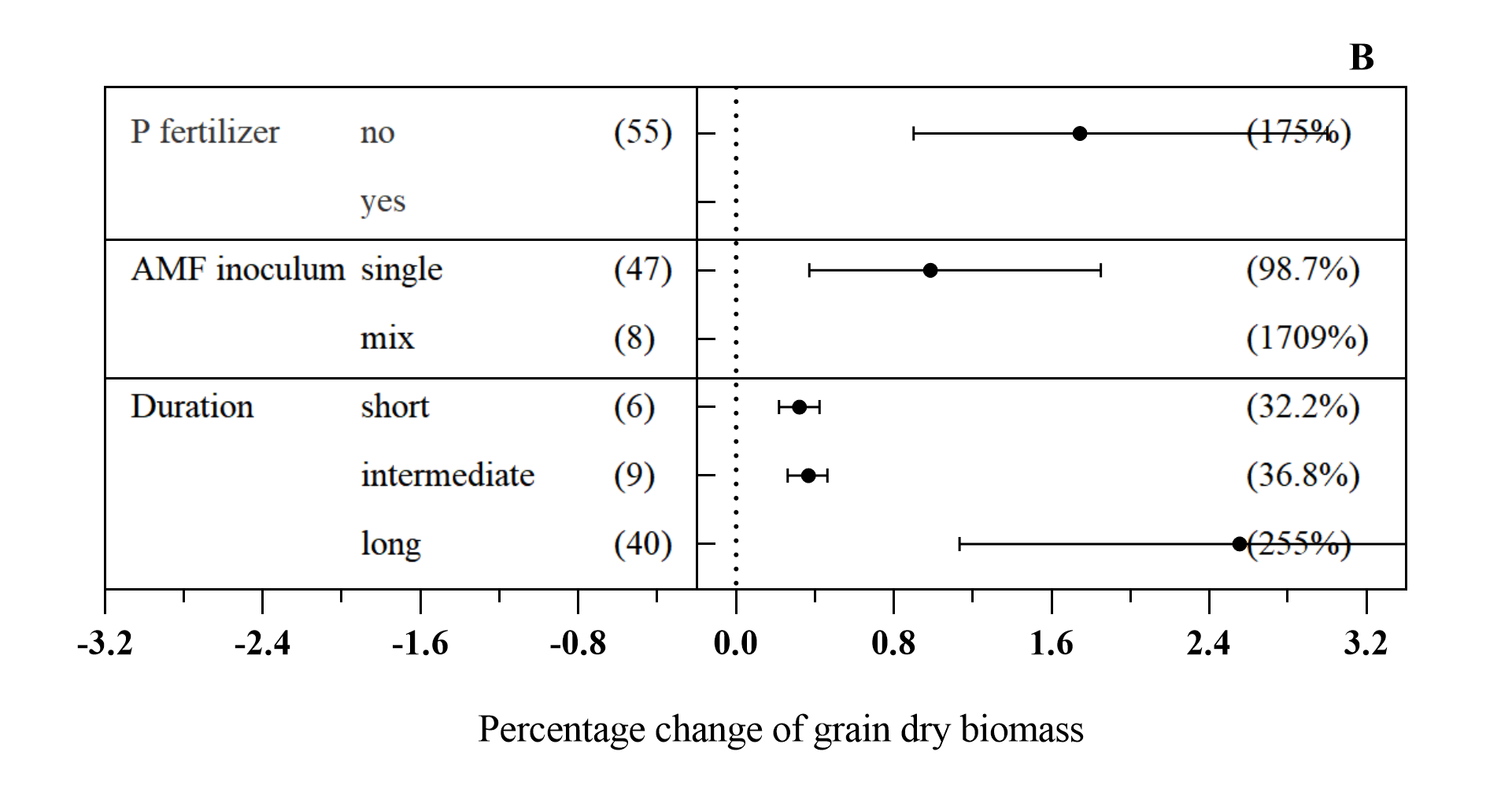

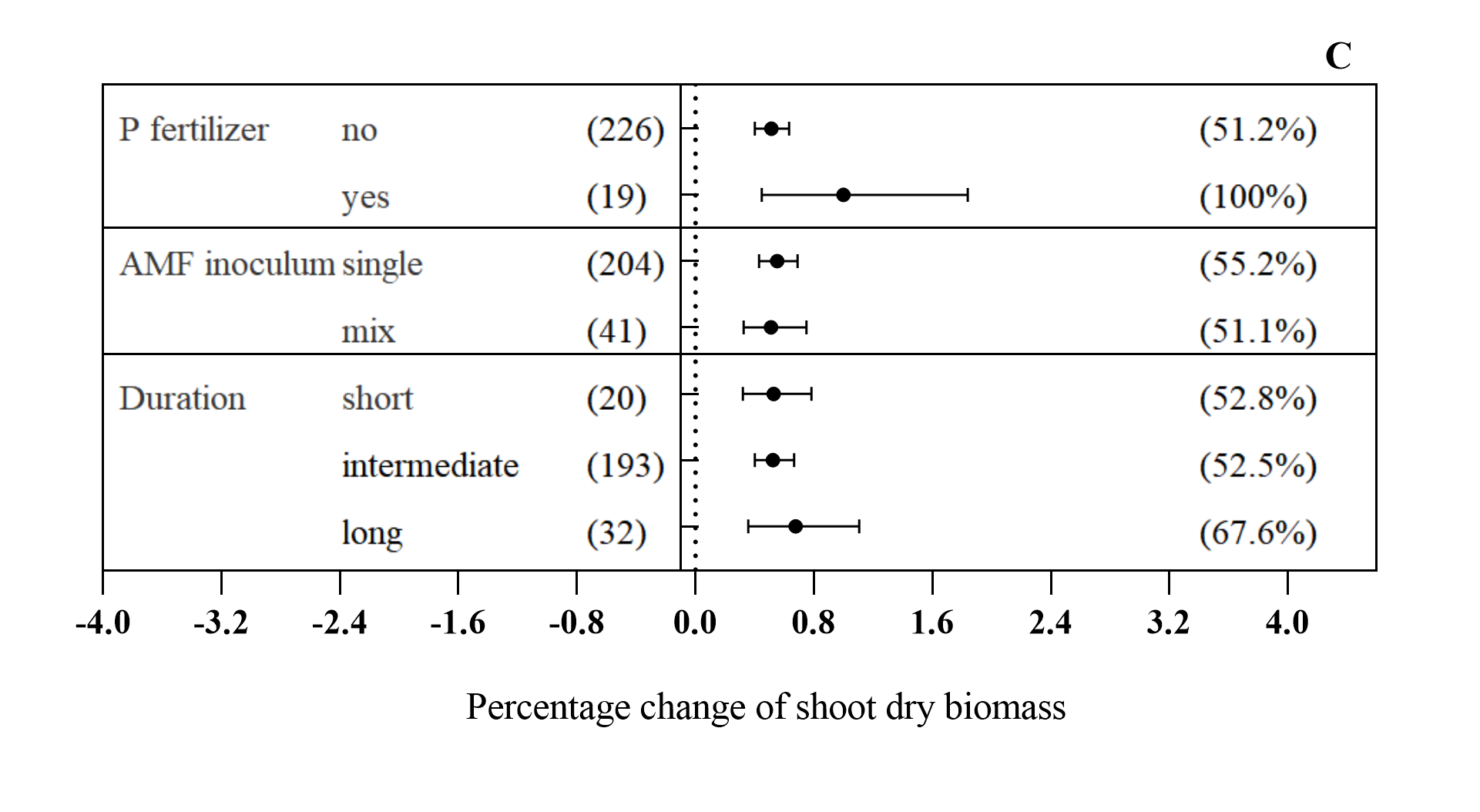

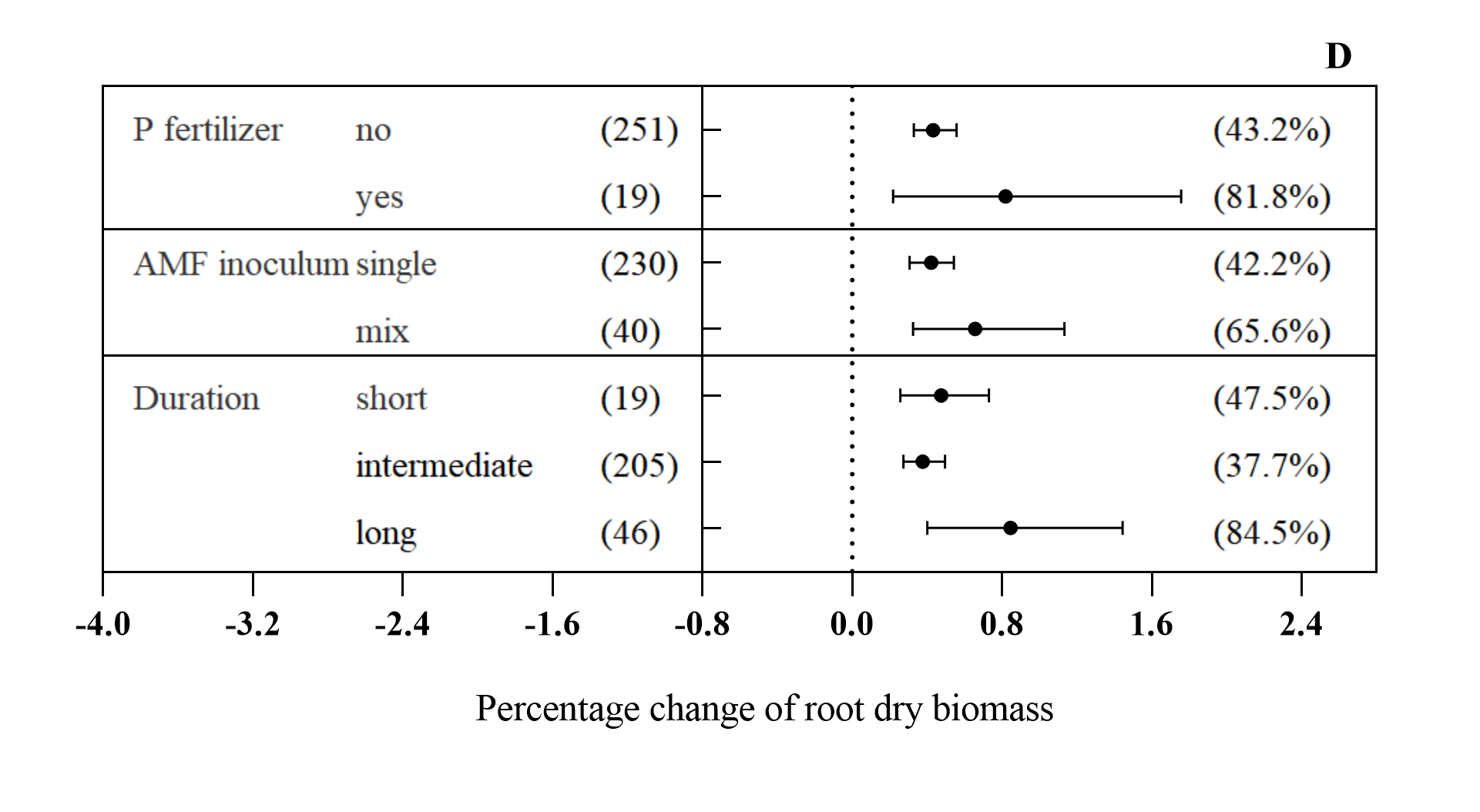


**Supplementary Figure 8.** Effects of mycorrhizal infection on P concentration in total plants (A), grains (B), shoots (C) and roots (D).


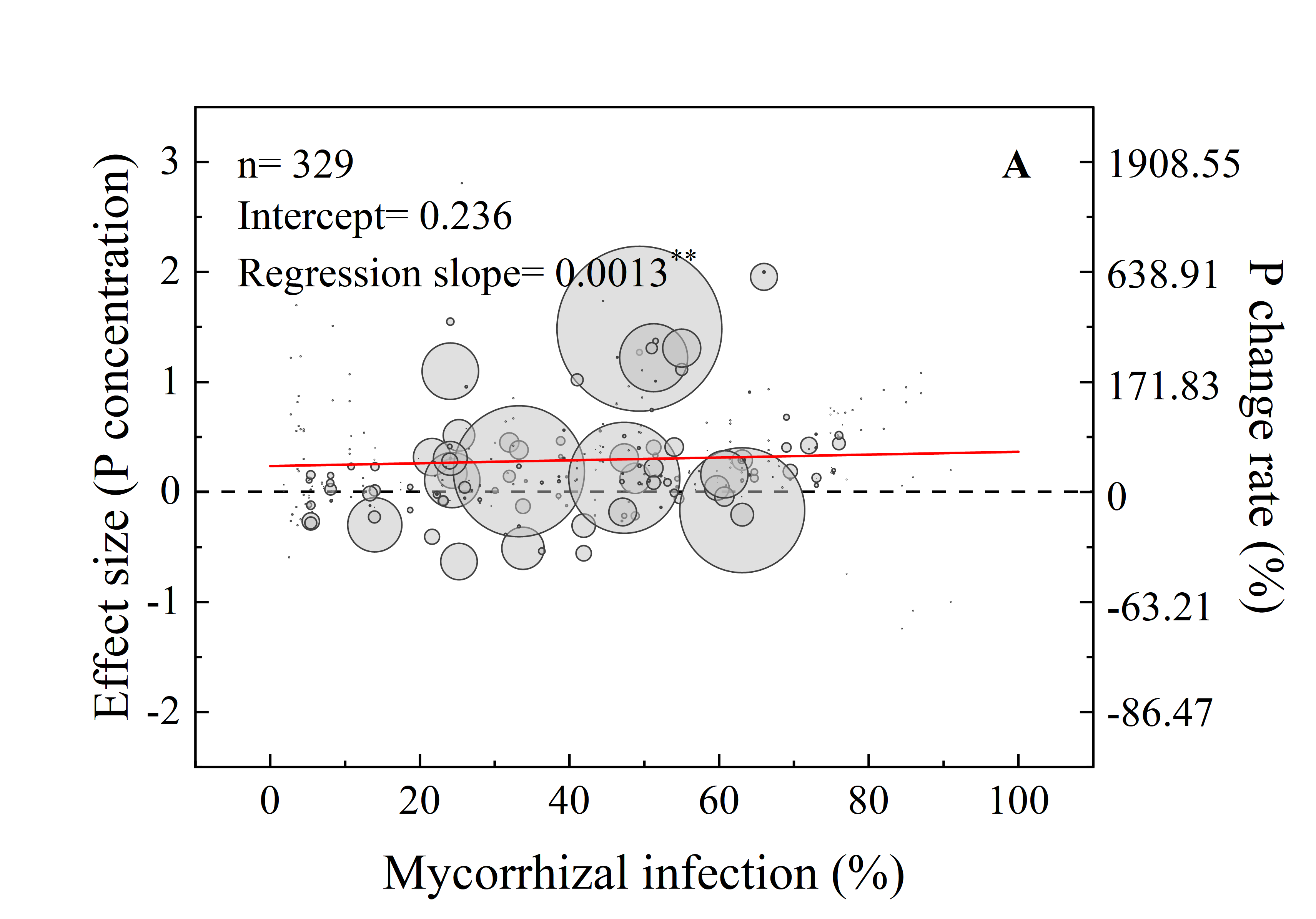

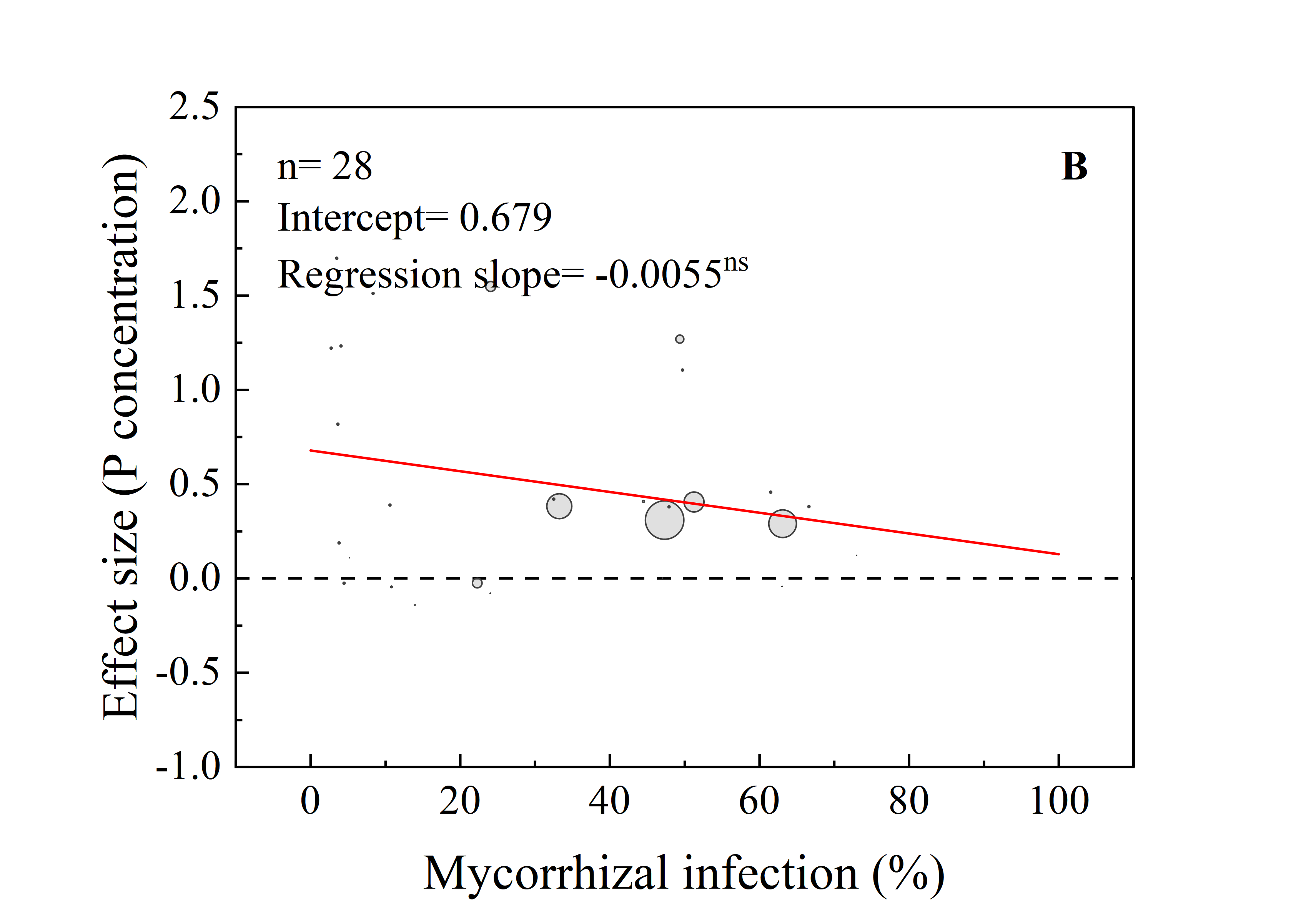

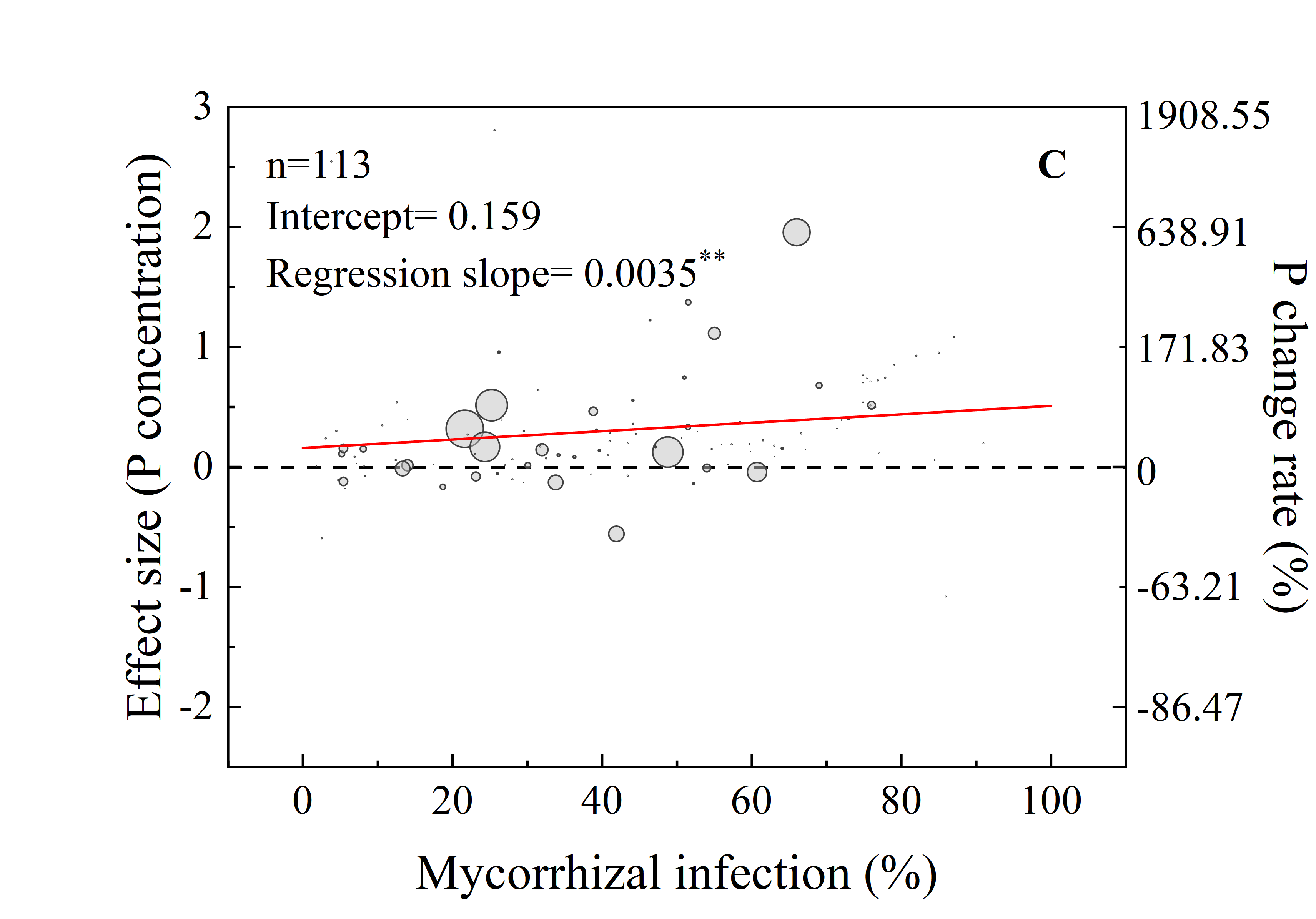

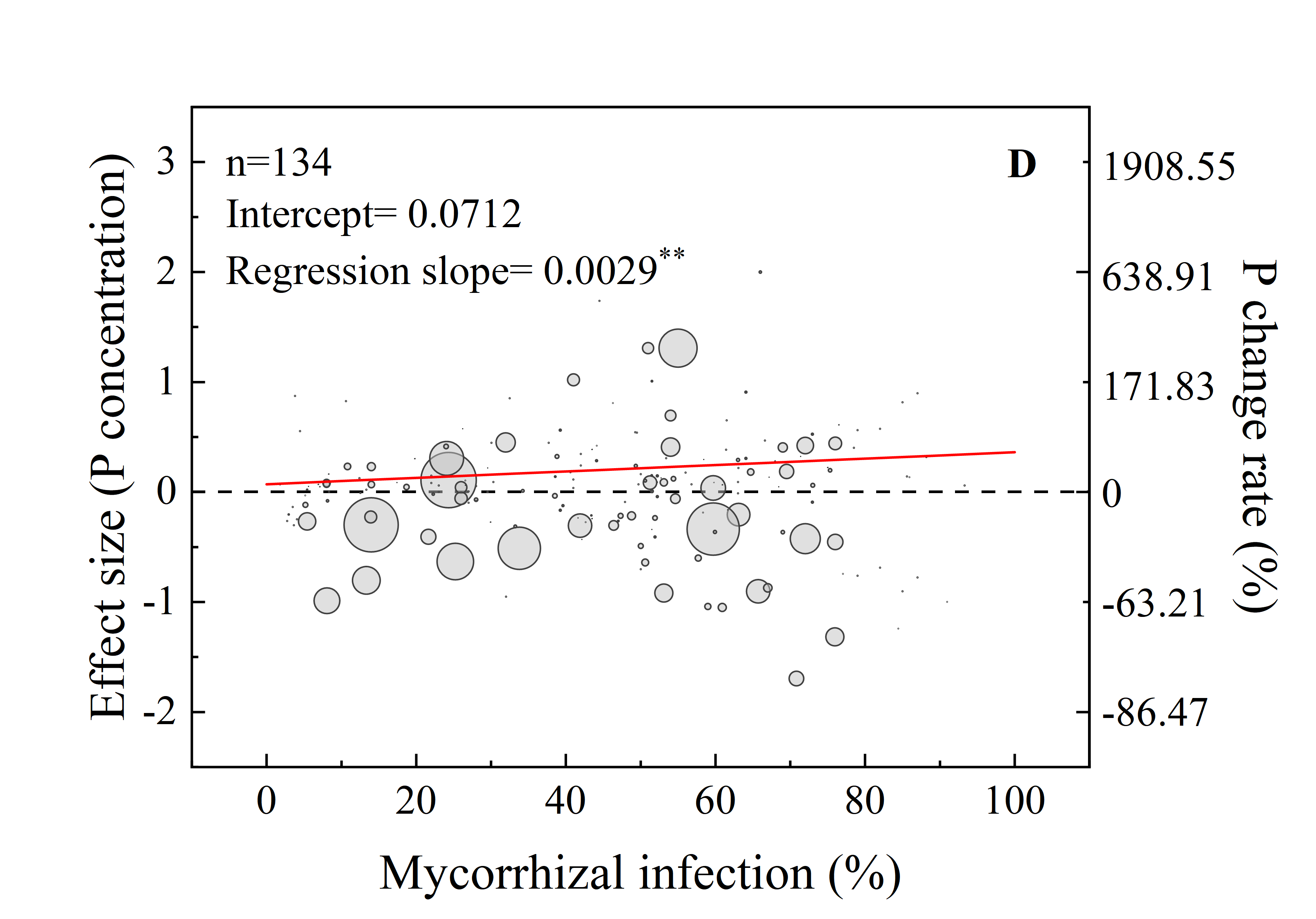

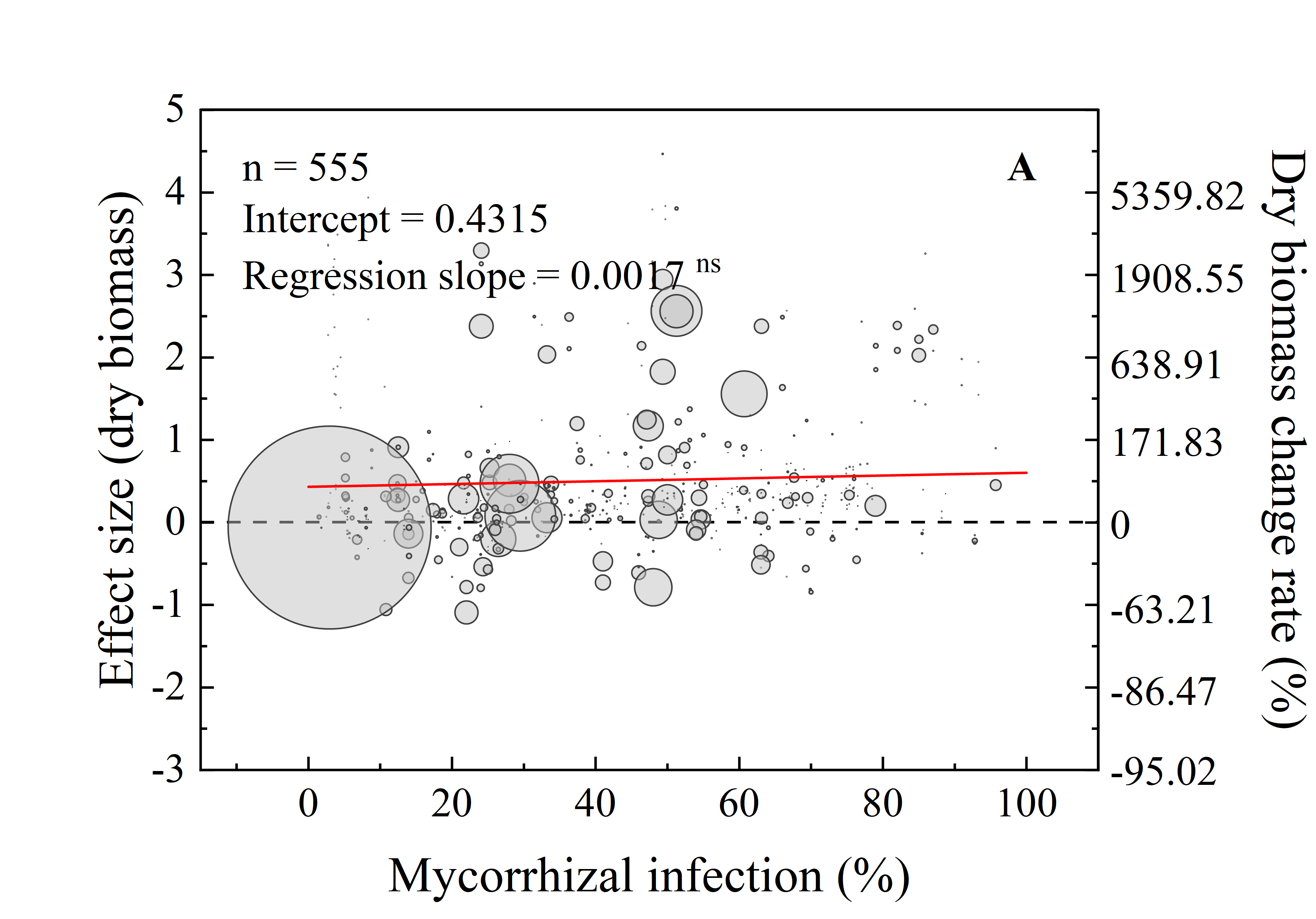

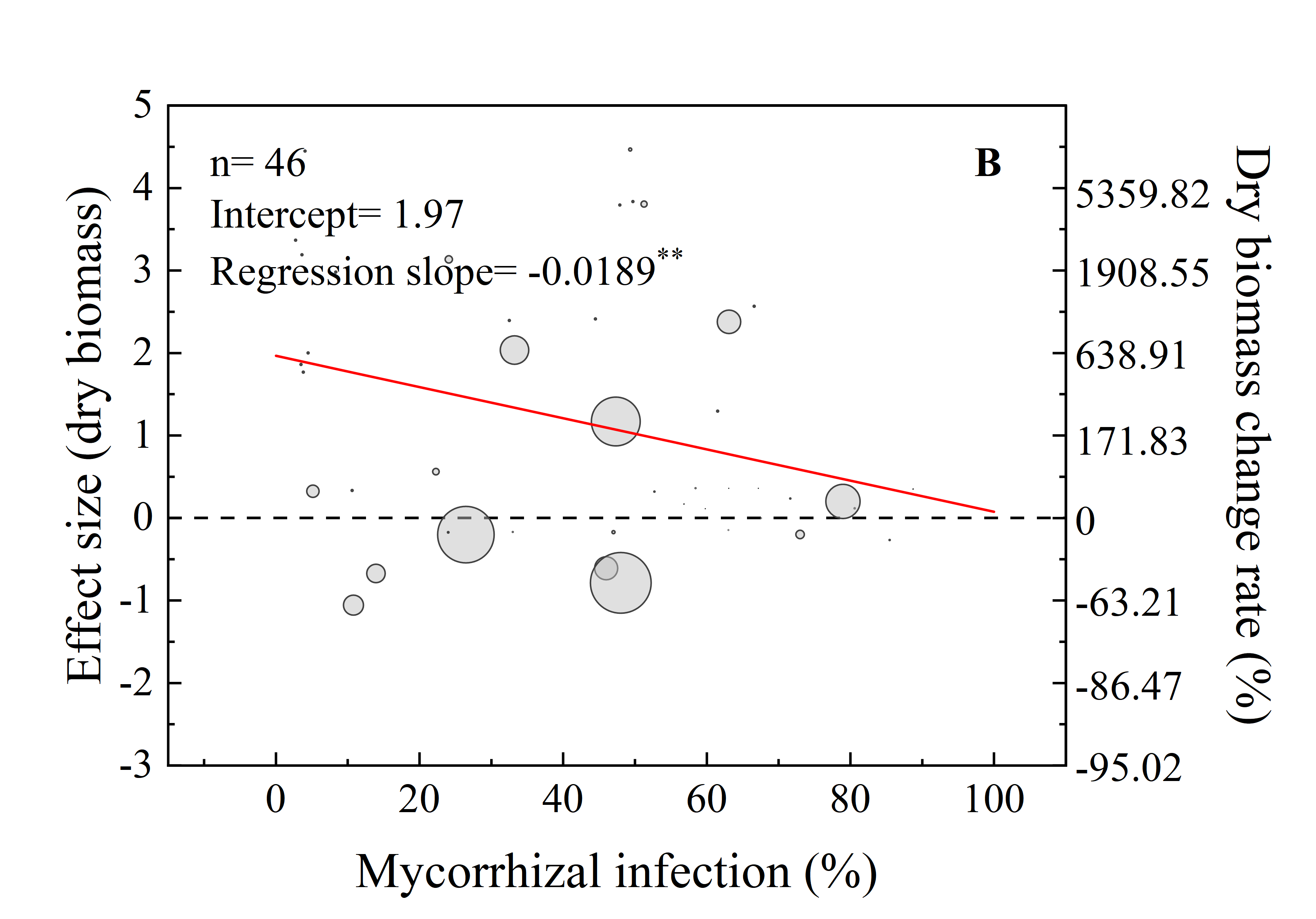

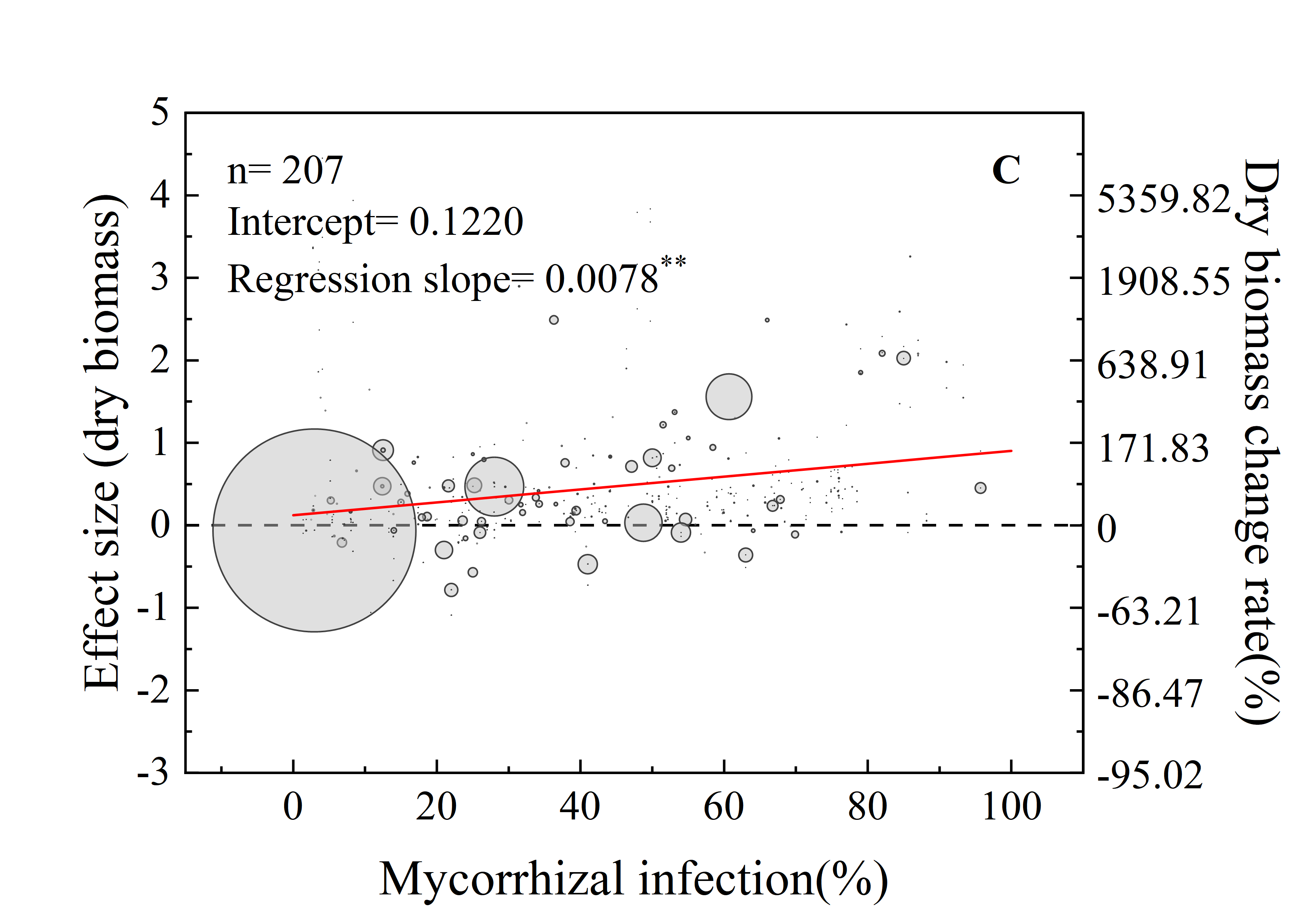

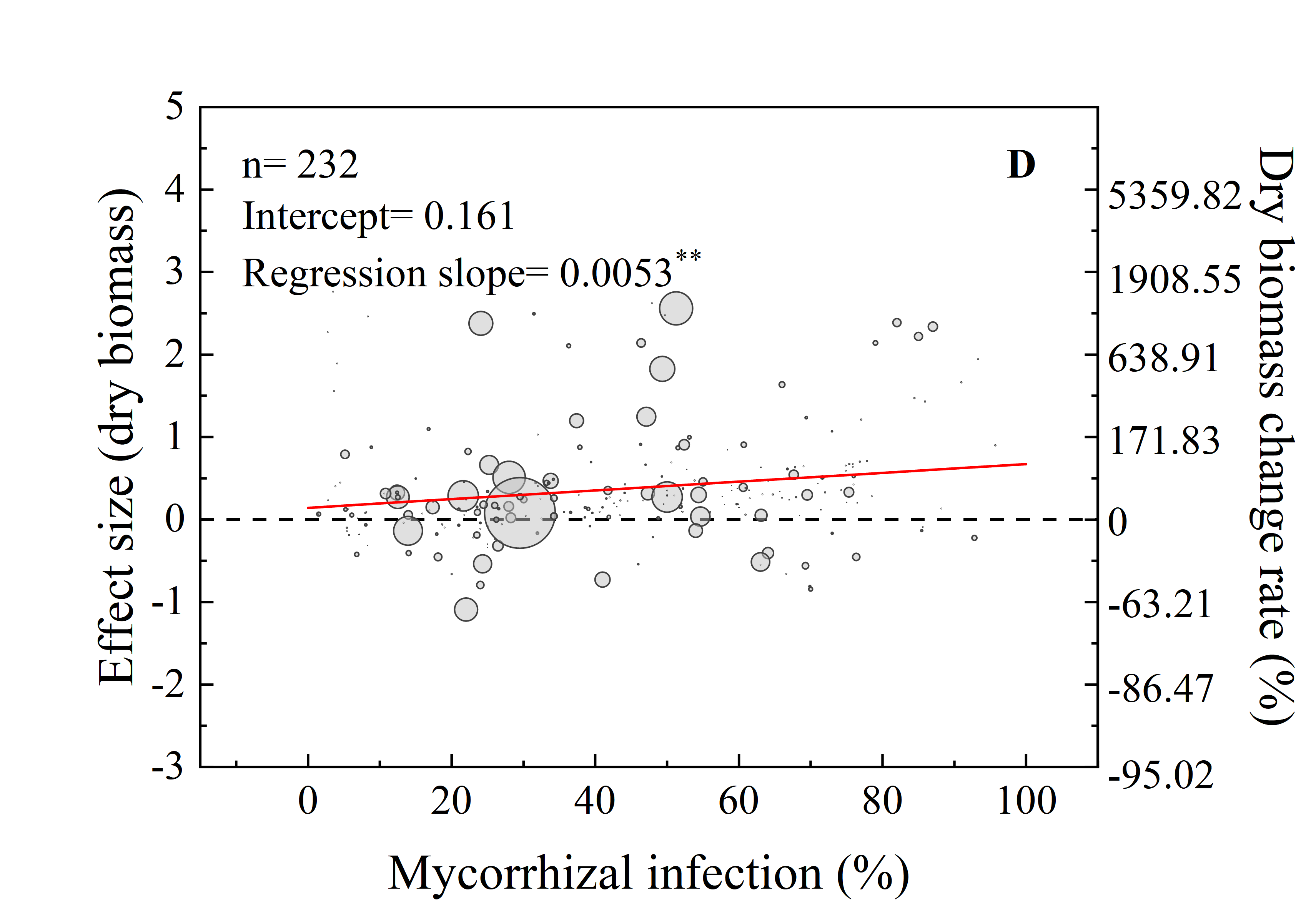


**Supplementary Figure 9.** Effects of mycorrhizal infection on dry biomass in total plants (A), grains (B), shoots (C) and roots (D).

## Supplementary Table

**Supplementary Table 1.** Data set heterogeneity test and publication bias test results.

| Index | As concentration dataset | P concentration dataset | Dry biomass dataset |
| --- | --- | --- | --- |
| n | 634 | 376 | 651 |
| Q_T_ | 74708 ^***^ | 26408^***^ | 100187^***^ |
| I^2^(%) | 99.2 | 98.6 | 99.3 |
| Fail-safe number | 218927 | 90118 | 152660 |

Number of observations (n); total heterogeneity (Q_T_); percentage of heterogeneity (I^2^); Fail-safe number. Significance of the Q_T_: ns; *; **; *** = not significant; significant at *P* < 0.05; significant at *P* < 0.01; significant at *P* < 0.001, respectively.
